# Defining depth requirements to conserve fish assemblages from water take in an intermittent river

**DOI:** 10.1038/s41598-024-81339-5

**Published:** 2024-12-02

**Authors:** Daniel C. Gwinn, Leah S. Beesley, Bradley J. Pusey, Michael M. Douglas, Chris S. Keogh, Oliver Pratt, Tom Ryan, Mark J. Kennard, Thiaggo C. Tayer, Caroline A. Canham, Lewis G. Coggins, Samantha A. Setterfield

**Affiliations:** 1 Biometric Research, South Fremantle, 6162 Australia; 2 School of Agriculture and Environment, The University of Western Australia, Perth, 6009 Australia; 3 Hillarys, 6025 Australia; 4 Australian Rivers Institute, Griffith University, Brisbane, 4111 Australia; 5 NOAA Fisheries, Southeast Fisheries Science Centre, Beaufort, NC USA

**Keywords:** Depth, Fitzroy River, Integrated species-distribution model, Fourth-corner solution, Incomplete detection, Barramundi, Ecology, Community ecology, Freshwater ecology

## Abstract

River systems once safeguarded from water development are being developed. This includes intermittent rivers that annually dry to a series of pools. Describing fish species relationships between abundance and pool depth can help managers set water-take rules that protect fish in dry-season pools. We sampled fish in main-channel and floodplain pools that spanned a gradient of depths and overcame sampling challenges by accounting for interacting effects of species mean length, environmental attributes, and sampling attributes on fish capture probabilities. Fish abundance-depth relationships varied systematically with species mean length, mesohabitat type (main channel, floodplain), water turbidity, and structural complexity, highlighting system complexity and the potential generality of abundance-depth relationships. Similarly, fish length moderated the effects of environmental attributes on capture probability for all sampling methods. We evaluated impacts of hypothetical water-take regulations on fish species’ distributions. Results suggested that water-take rules prohibiting draining of main-channel pools below 1.65 m and reducing floodplain pools by no more than 14% minimises impacts to species’ distributions, promoting conservation of the fish community. Additionally, our approach demonstrates the capacity of species length for predicting distributional and sampling patterns of fish species.

**Supplementary Information:**

The online version contains supplementary material available at 10.1038/s41598-024-81339-5.

## Introduction

Conservation of freshwater resources is a global concern and is increasing as a policy priority at the international level^1,2^. River systems once safeguarded from water development due to remote location, unreliable flow, environmental value, or cultural significance are now being developed^3,4^. Past failures of policy to balance the functional needs of freshwater ecosystems with water development have resulted in catastrophic ecological effects across the planet e.g., Grand Canyon^5^, Yangtze River^6^ and Murray-Darling Basin^7^. Today, growing awareness of the susceptibility of freshwater systems and our past failures to effectively manage them^8–10^, has increased societal pressure to ensure that new developments are sustainable^11^.

In northern Australia there is increasing pressure to extract water from remote rivers for agricultural production^12^. The Fitzroy River in the Kimberly region of Western Australia is an example of one such system. This tropical intermittent river has limited access and high inter-annual flow variability posing challenges for water resource development^12^. The river is recognised locally, nationally, and internationally for its important environmental and cultural values that relate closely to the biodiversity of the system^13^. Recently, the state government has received proposals for water extraction and is developing policy to protect the cultural and ecological services^14^. However, creating sustainable water-take rules that support Indigenous values, ecological functioning, and societal needs is a difficult process due to differing world views between Indigenous peoples and western settlers^15^, and a deficiency of rigorous research needed to inform policy trade-offs^16^.

Disconnected dry-season pools represent a distinct feature of intermittent rivers. Isolated pools can act as refuges, supporting recolonization of fish assemblages during rewetting^17^. The depth of pools and correlated features (e.g., pool volume, pool persistence, water quality, temporal stability) can influence competition, predation, and mortality^18,19^, structuring fish assemblages available for recolonization and recruitment^17^. Alternatively, disconnected pools can act as population sinks when water depths in pools drop below critical levels before seasonal rewetting occurs^20,21^. Understanding relationships between the local fish assemblages and water depths of pools could provide an important and accessible guide for managers to consider when setting water take rules. In northern Australian rivers, past research has identified general ecological patterns across depth gradients of pools e.g.^22–24^, but the transferability of these patterns to the Fitzroy River remains uncertain. Thus, to support water policy for the Fitzroy River, specific depth relationships need to be defined. Such relationships will assist decision makers to discriminate between competing policies options and formalize policy trade-offs^25^.

The primary challenge faced when describing patterns in fish distribution across water depths is that observed variation in the fish assemblage can be confounded by the sampling effects. Fish assemblages are known to partition according to water depth with smaller species more common in shallower waters and larger species more common in deeper waters^26,27^. This can be problematic when drawing inference from catch data because different sampling methods are required to effectively sample different depths and inherently select for different sized individuals and species^28,29^. These confounding processes can be difficult to disentangle and when ignored can lead to spurious conclusions, a lack of transferable patterns, and suboptimal management.

The objective of our study was to describe species-specific patterns in fish distribution in relation to pool depth to guide water policy. To disentangle fish distributions from the sampling process, we developed an integrated hierarchical multi-species-distribution model that separates patterns in fish abundance and capture probability with depth and other environmental covariates. To demonstrate how fish-depth relationships could inform management decisions we used our fitted model to predict the outcomes of water take rules applied to two hypothetical water take scenarios. The findings of our study contribute to flow-ecology relationships directly informing water-take rules for sustainable development of the Fitzroy River, Western Australia. More broadly, our study increases knowledge of fish-depth relationships in intermittent rivers and the potential for knowledge transfer to other similar systems.

## Methods

### Study system and context

**Fig. 1:**
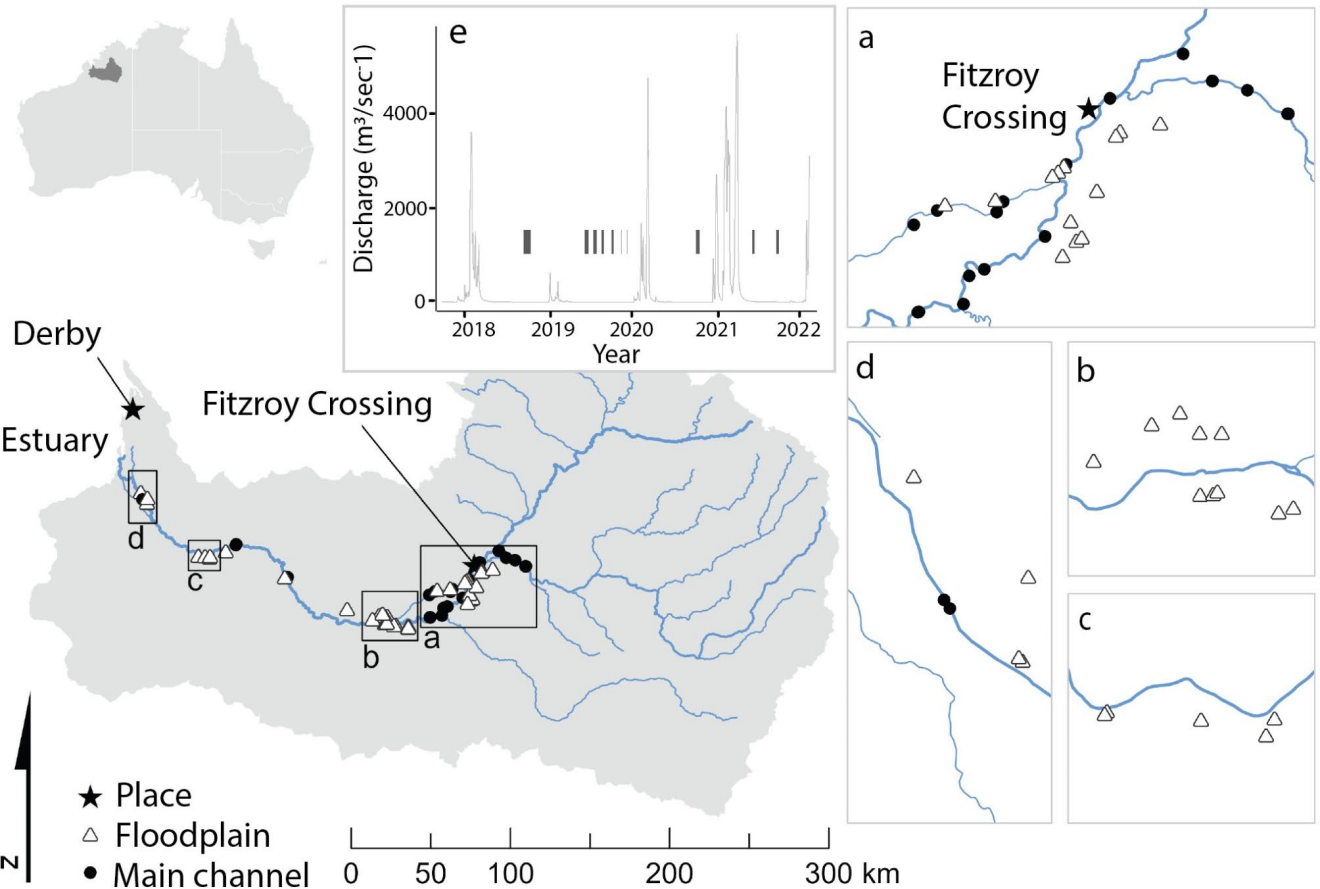
The location of main channel and floodplain pools surveyed for fish (**a–d**) and hydrograph (**e**) during the survey period for the Fitzroy River, Kimberley, Western Australia. Map created using ARCGIS Pro (version 3.3).

The Fitzroy River is ~ 700-km long and located within the wet-dry tropics savannah landscape of Western Australia ‘s Kimberley region. The wet season is predictable (November–March) with considerable annual variation in magnitude^12^ (Fig. 1e). Flow is highly intermittent, but predictable during the Austral summer (November to March) wet season (predictable summer highly intermittent class 10^30^). These strong seasonal and inter-annual flow dynamics create periods of extremely high and low water availability. The river is Heritage listed and supports considerable biodiversity, including 37 fish species^31^, several of conservation significance that are listed by the IUCN, including the freshwater sawfish (*Pristis pristis*). No alien species have been reported to date.

The study targeted the lower reaches of the Fitzroy River which are considered most likely for future water extraction. The main channel is sinuous with alternating deep and shallow reaches^32^. Reaches are connected facilitating animal passage during the wet season and reduced to isolated pools during the dry season. Pools are typically several kilometres long (30-500-m wide) and are sustained by regional and alluvial groundwater^32^. The floodplain is dissected by distributary creeks that disperse water from the main channel during elevated within-bank flows^32^.

### Study design and fish sampling

We sampled fish in pools accessible by 4-wheel drive vehicles during dry conditions between June and December 2018–2021. This included 20 main-channel and 39 floodplain pools (Fig. 1a–d). Pools were sampled once a year, with many sampled on multiple years, totalling 107 site-visit combinations. Total wet season flows were 1606, 235, 1293, and 4046 gigalitres prior to sampling in 2018–2021 respectively. During each pool visit, fish were sampled with boat electrofishing, backpack electrofishing, large-mesh seine, and/or small-mesh seine. The choice of method was dictated by the conditions of the site and risk of saltwater crocodile attack; however, we used multiple methods when possible. All sampling replicates were spatially distinct within each pool and targeted homogenous units of all available habitats (e.g., depth, substrate, and structural complexity) to maximizing capture opportunities across fish species and avoid correlation between site-level and sample-level environmental covariates (particularly depth).

Boat electrofishing was used in pools typically > 1-m deep while backpack electrofishing fishing unit was used predominantly in shallow, clear-water habitats. A 10-m or 5-m long, 9-mm mesh beach seine (large-mesh seine) was used depending on pool size and complex structure, while a 7-m long, 2-mm mesh beach seine (small-mesh seine) was used to target smaller fish. Total number of samples collected per site visit ranged between two and 18. Sampling effort for each replicate was recorded as seconds of power for electrofishing and length of haul for seines. Fish collected by all methods were held in aerated buckets, counted, identified to species, and released. The first 50 individuals of each species and each gear type were measured (standard length, mm) and weighed on a 500 g–5 kg balance as appropriate. Detailed sampling information is available in the Supplement.

### Animal use statement

Research was carried out under Fisheries exemptions #191-2009-27 and 2974, and The University of Western Australia’s Animal Ethics permits RA/3/100/884 and RA/3/100/1536 (Animal Ethics Committee, The University of Western Australia). All researchers conducting field work had a valid Permission to Use Animals (PUA) licence to use animals for scientific purposes as per the Animal Welfare Act 2002 (Western Australia). All field procedures were performed in accordance with relevant guidelines and regulations, including the ARRIVE guidelines.

### Environmental variables

We recorded environmental variables at the spatial scale of the pool and the spatial scale of the sample location to discriminate between the potential influences on species distributions and species capture probabilities. Variables recorded at the pool scale included mean depth, maximum depth, water turbidity, percent complex structure, electrical conductivity, pH, dissolved oxygen, percent composition of substrate, mean wetted width, and length. Variables recorded at the sample scale included mean depth, maximum depth, and percent complex structure. Percent structural complexity was defined as the percent cover of all complex habitats, including large and small woody debris, roots, undercut banks, mud boulders, and macrophyte beds. Methodological details are provided in the Supplement.

### Modelling approach

**Fig. 2:**
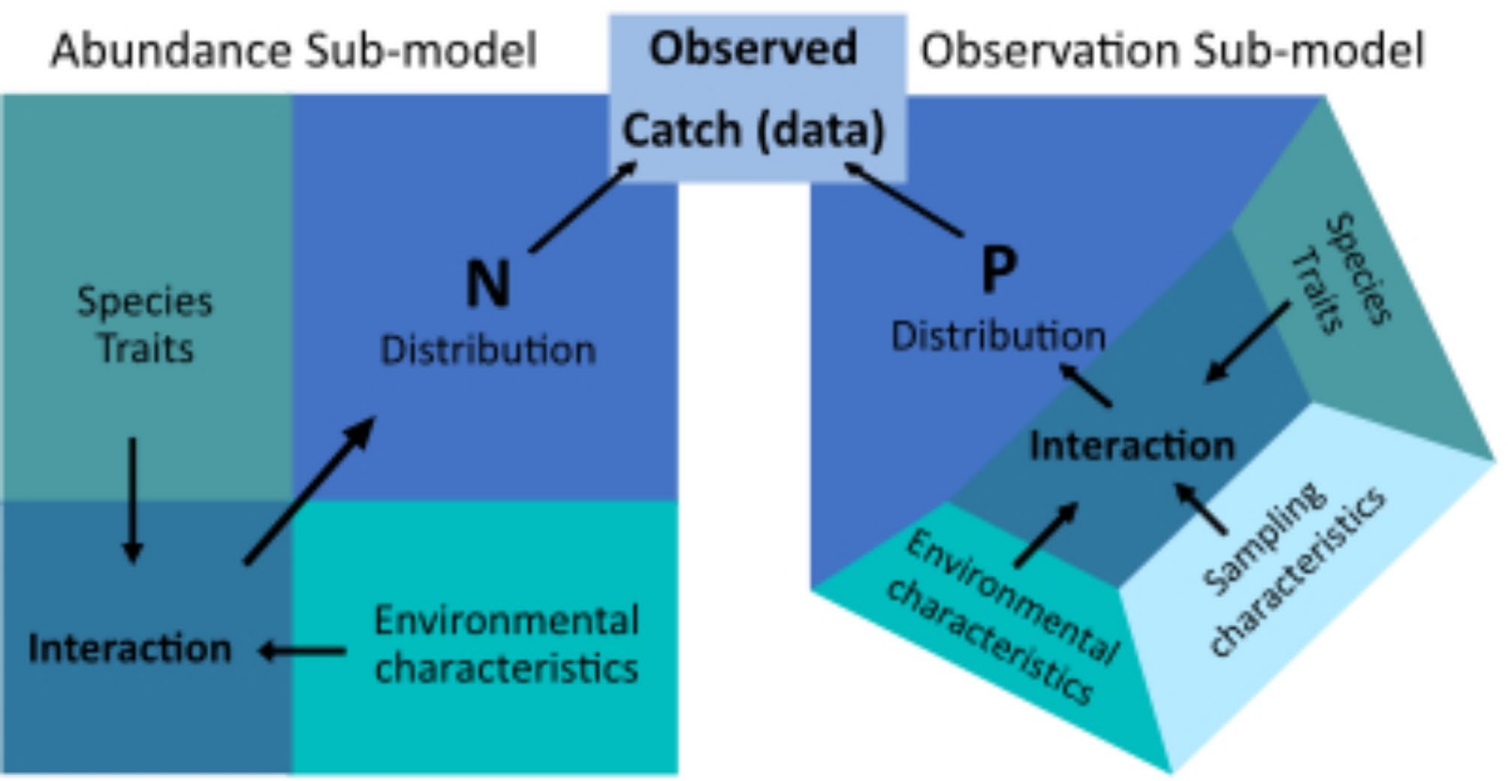
A generalized diagrammatic representation of our fourth-corner approach to species abundance and observation sub-models. The ecological model describes species traits interacting with environmental characteristics to determine the distribution of species abundance (N; i.e., Fourth-corner solution, Legendre et al.^33^). The observation model describes species traits, environmental characteristics, and sampling characteristics interacting to determine the species-specific capture probability, denoted as P, of a sample. The intersection of the ecological and observation models results in the observed catch and composition of species in a sample, i.e., data.

Our model building strategy was to choose structures and covariates relevant to the management question that were realistic, generalizable, and well-justified in the literature. We developed a novel integrated hierarchical multi-species distribution model with several strategic features. The model was composed of several hierarchical layers including an abundance sub-model describing the latent distribution of species abundance. We integrated data across sampling methods by specifying unique observation sub-models for each method. To aid in disentangling the confounding effects of environmental variables on abundance and capture probability, we adopted the strategy of “the fourth corner solution”^33^ by explicitly modelling species-specific environmental effects as emergent properties of the interaction between fish length and environmental variables to describe patterns in species abundance and capture probability. A diagrammatic representation of our model is depicted in Fig. 2.

### Model and likelihood

The model describes pool-scale species abundances as latent random variables conforming to a Poisson distribution as, ${N}_{i,j}\sim\text{P}\text{o}\text{i}\text{s}\text{s}\text{o}\text{n}\left({\lambda }_{i,j}\right)$, where ${\lambda }_{i,j}$ is the expected abundance of species *i* in pool *j*. The model likelihood assumes that binary detection/non-detection data result from a Bernoulli process as, ${y}_{i,j,k}\sim\text{B}\text{e}\text{r}\text{n}\text{o}\text{u}\text{l}\text{l}\text{i}\left({p}_{i,j,k}\right)$, where ${p}_{i,j,k}$ is the probability of detecting at least one individual of species *i*, at pool *j*, in sample *k* (i.e., detection probability). We linked the abundance model to the likelihood by specifying the detection probability as, ${p}_{i,j,k}=1-{\left(1-{r}_{i,j,k}\right)}^{{N}_{i,j}}$, where ${r}_{i,j,k}$ is the proportion of the population captured in each sample *k* (i.e., capture probability^34^). This equation formalizes the assumption that all individuals are independent relative to capture (i.e., independent binomial samples) which will be violated to an unknown extent due to the interaction between spatially replicated sampling and aggregating behaviours of fish within pools. Although this violation is expected to result in biased estimates of both *N* and *r*, Gwinn^35^ demonstrated robust covariate effect estimates of abundance and capture probability across a broad range of aggregation levels. Thus, *N* estimates must be interpreted as relative abundance that is corrected for variable capture probability as opposed to absolute abundance, which is acceptable for our purpose. Furthermore, alternative methods for estimating fish distributions with heterogeneous counts, demonstrate high biases and poor fitting behaviours, making this approach a practical choice^36^.

### Species-abundance sub-model

We structured the abundance sub-model to conform to the reality that at zero water depth, the abundance of all species was zero and increased with depth to a potential asymptote or declined past an optimal depth. We adopted a Ricker model^37^ to describe this pattern, as this structure can describe species favouring shallow or deep waters, with or without an optimal depth beyond which abundance is constant or declines. We strategically included water turbidity and structural complexity of each pool as covariates as we expected a likely influence on fish abundance. We included interaction effects of mesohabitat with depth and environmental covariates to account for different processes affecting abundance in these distinct river habitats. Thus, we expressed the abundance of species $i$ at pool $j$ as,

1 $$\begin{aligned}\text{l}\text{o}\text{g}\left({\lambda }_{i,j}\right)&=\left({\beta }_{1,i}+{\beta }_{3,i}{M}_{j}\right)+\text{l}\text{o}\text{g}\left({D}_{j}\right)-{e}^{\left({\beta }_{2,i}+{\beta }_{4,i}{M}_{j}\right)}{D}_{j}+{\beta }_{5,i}{T}_{j}+{\beta }_{6,i}{T}_{j}{M}_{j}\\& \quad+{\beta }_{7,i}{S}_{j}+{\beta }_{8,i}{S}_{j}{M}_{j}+{\beta }_{9,i}{R}_{j} +{\beta }_{10,i}{R}_{j}{M}_{j}+{\epsilon }_{j}^{pool}+{\epsilon }_{j}^{time},\end{aligned}$$

where ${\beta }_{1,i}$ and ${\beta }_{2,i}$ describe the relationship of abundance with water depth (${D}_{j}$) in the main channel for species $i$. The parameters ${\beta }_{3,i}$ and ${\beta }_{4,i}$ allow for the estimation of different patterns in abundance with depth in the floodplain mesohabitat (indexed by vector ${M}_{j}$ where 0 = main channel and 1 = floodplain). The parameters ${\beta }_{5,i}$, and ${\beta }_{6,i}$ model effects of turbidity (${T}_{j}$) and interactions with mesohabitat, while parameters ${\beta }_{7,i}$ and ${\beta }_{8,i}$ model the effects of structural complexity (${S}_{j}$) and interactions with mesohabitat. The parameters ${\beta }_{9,i}$ and ${\beta }_{10,i}$ model the potential influences of river km (${R}_{j}$) on abundance and interactions with mesohabitat. We included a pool and sample-time random effects ${\epsilon }_{j}^{pool}$ and ${\epsilon }_{j}^{time}$to allow for extra-Poisson variation in abundance and account for resampling of pools. The depth covariate ${D}_{j}$ was scaled to a maximum value of one to accommodate the Ricker model form, and ${T}_{j}$, ${S}_{j}$, and ${R}_{j}$ were centered on zero and scaled to one standard deviation to facilitate the fitting process and prior specification.

### Observation sub-model

A substantial body of literature exists describing patterns in capture probability of freshwater fish. We used this literature to develop expectations of major drivers of variation in capture probability for each sampling method to determine a realistic and parsimonious model parameterization. Based on this literature, we expected either a monotonic or dome-shaped pattern of catchability ($q$, proportion of vulnerable population captured with one unit of sampling effort) with water depth at the sample location due to interactions between variable depth preferences of fish species^28,29^, and decreasing electrofishing electrical fields and netting efficiencies with depth^38^. Similarly, we expected electrofishing catchability to display a dome-shaped relationship with turbidity, with increasing catchability as turbidity increases due to more adventurous fish behaviour, followed by a decline in catchability due to reduced netting efficiency^39^. We expected complex structure, such as aquatic vegetation and woody debris, to have either have no effect or a positive or negative monotonic effect on catchability due to interactions between variable fish habitat preferences with increased difficulty netting and seining fish as complexity increased^40^. The possible effects of water conductivity were only modelled for electrofishing sampling methods, as efficiency of the electrical field is optimized when the conductivity in the environment is equivalent to the internal conductivity of fish^41^, both of which are expected to vary. Lastly, we considered a range of possible functional forms to describe the relationship between capture probability and sampling effort, which included proportional, saturating, and nil, all of which have been reported in the literature due to spatial aggregating of fish, time measurements of sampling effort, and gear saturation^42^. To accommodate for these potential effects, we expressed our observation sub-models for each sampling method as,

2 $$\text{l}\text{o}\text{g}\left({q}_{i,j,k}\right)={\phi }_{1,i}+\text{log}\left({\acute{D}}_{j,k}\right)-{e}^{{\phi }_{2,i}}{\acute{D}}_{j,k}+{\phi }_{3,i}{T}_{j}+{\phi }_{4,i}{T}_{j}^{2}+{\phi }_{5,i}{\acute{S}}_{j,k}+{\phi }_{6,i}{C}_{j},$$

3 $${r}_{i,j,k}={E}_{j,k}^{\upsilon }\left(1-{e}^{-{e}^{{q}_{i,j,k}}}\right),$$

where ${q}_{i,j,k}$ in Eq. (2) represents the catchability of fish species *i* in pool *j* and sample *k*. The parameters of Eq. (2), ${\phi }_{1,i}$ and ${\phi }_{2,i}$, model the pattern of catchability with sample-scale depth (${\acute{D}}_{j,k}$, mean depth of electrofishing transect and maximum depth of seine haul); the parameters ${\phi }_{3,i}$ and ${\phi }_{4,i}$ model the relationship of catchability to pool turbidity (${T}_{j}$); and the parameters ${\phi }_{5,i}$ and ${\phi }_{6,i}$ model the potential influence of sample-scale complex structure (${\acute{S}}_{j,k}$) and pool conductivity (${C}_{j}$, only in electrofishing sub-models). Equation (3) scales catchability ${q}_{i,j,k}$ by sampling effort to derive capture probability ${r}_{i,j,k}$. The term ${E}_{j,k}^{\upsilon }$, is a flexible function that describes the relationship of ${r}_{i,j,k}$ to effort, where the variable ${E}_{j,k}$ is the sampling effort scaled to range between zero and one, and parameter $\upsilon$ determines the shape of the relationship between ${r}_{i,j,k}$ and ${E}_{j,k}$. Values of $\upsilon =1$ describe a proportional relationship, values < 1 describe a saturating relationship, and values of $\upsilon =0$ describe no relationship. For the large-mesh seine we included an estimated scaling parameter to account for the expected reduction in capture probability of the 5-m relative to 10-m seine length as,

${r}_{i,j,k}={\varsigma E}_{j,k}^{\upsilon }\left(1-{e}^{-{e}^{{q}_{i,j,k}}}\right)$, where $\varsigma$ is a parameter with potential values from 0 to 1 that scales the ${r}_{i,j,k}$ for 5-m seine samples.

### Species-length covariate

Species-level parameters in the abundance and observation sub-models, ${\beta }_{1-10,i}$ and ${\phi }_{1-6,i}$, were modelled as random effects across species with means described as linear fixed effect of species average length. We chose to model the potential influence of fish length because of its inherent relationship to depth preferences^28,43^, habitat preferences^28,29^, and vulnerability to electrofishing and mesh-based sampling^41,44^. Thus, we expected that fish length would likely interact with environmental covariates and sampling methods, providing a powerful predictor of distributional and capture probability patterns increasing the predictive performance and generality of our model^45,46^. We specified species-level parameters as random effects conforming to normal distributions as,

4 $${\beta }_{s,i} \& {\phi }_{s,i}\sim\text{N}\text{o}\text{r}\text{m}\text{a}\text{l}\left({\mu }_{s,i},{\sigma }_{s}^{2}\right),$$

5 $${\mu }_{s,i}={\eta }_{1,s}+{\eta }_{2,s}{L}_{i},$$

where ${\mu }_{s,i}$ is the component of the ${\beta }_{1-10,i}$ and ${\phi }_{1-6,i}$ explained by fish length ${L}_{i}$ and ${\sigma }^{2}$ is the variance describing the random component of the parameter effect in Eq. (4). The parameter ${\mu }_{s,i}$ is described as linear model with intercept ${\eta }_{1,s}$ and slope (i.e., length effect) ${\eta }_{2,s}$ (Eq. 5). This formulation allows the covariate effects for individual species in the abundance and observation sub-models to diverge from the potential relationship with fish size, allowing full flexibility of the model to account for plausible variation in all covariate effects and reducing the likelihood of spurious outcomes^47^. Model code and further description is presented in the Supplement.

### Model fitting and regularization methods

Posterior probability distributions of model parameters were sampled using a Gibbs sampler implemented in JAGS^48^ called from within program R with the library R2jags (http://mcmc-jags.sourceforge.net). Inference was drawn on four Markov Chain Monte Carlo (MCMC) chains with sufficient iteration to ensure convergence and minimum effective sample size > 400. To avoid overfitting and promote efficient mixing of MCMC chains, we specified mildly informative priors and mild regularization using a Bayesian penalized likelihood approach (see Supplement for full details).

### Interpretation for management

To illustrate how fish-depth relationships could assist the development of water policy, we applied our fitted model to predict the outcomes of two management scenarios. Scenario-1 investigated the conservation outcome of regulating the direct pumping of water from pools by setting a minimum depth rule that constrains water extraction to pools deeper than the target depth; when the pool depth is reduced to the target depth, extraction ceases. We evaluated minimum depth rules from 0.5 to 2.0 m. In the Fitzroy River, pools large enough to produce yields meaningful for irrigated agriculture are predominantly situated in the main channel; thus, we applied this management scenario to main channel pools only. Scenario-2 investigated the conservation outcome of regulating water extraction via floodplain harvesting. Floodplain harvesting is when water flowing through floodplain distributary creeks is collected and stored in excavated tanks (farm dams) for later use. Because floodplain harvesting affects the amount of water transported to collections of pools, we investigated a rule that would limit the average percent reduction in pool depths. We considered water extraction rules that would limit the reduction of floodplain pool depths from 0 to 90%. In practice, water extraction from main-channel pools and floodplain distributary creeks will be limited by economic trade-offs related to access and reliability of water sources, which we cannot predict. Thus, to simplify the problem and provide general guidance, we predicted the outcomes of the regulations of scenario-1, and 2 on the fish assemblages of the main-channel and floodplain pools sampled for this study, respectively. These pools are broadly representative of the landscape, providing a useful stylized illustration of expected conservation trade-offs and outcomes. To simulate the effects of the water extraction rules, we reduced the observed depths of main-channel pools to the minimum depth rule of scenario-1 and floodplain pools by the percent reduction rule of scenario-2, representing full extraction of the legal amount of water allowed by the hypothetical regulations. We then applied our model to predict the number of pools occupied by each fish species to represent the breadth of distribution. Lastly, we calculated the percent reduction of each species’ distribution for each management rule relative to the distribution predicted under no water extraction.

## Results

A total of 53,116 fish were caught, encompassing 21 species; 20 of which were found in main channel pools and 19 in floodplain pools (Supplementary Table S1). Rare species, i.e., those present in the river but caught at two or less sampling events, such as *Anguilla bicolor*, *Lutjanus argentimaculatus*,* Selenotoca multifasciata*,* P. pristis*, *Carcharhinus leucas* and *Urogymnus dalyensis* were omitted from the above summary and from all analyses. Sampled pools spanned a gradient of maximum depth from 0.35 to 4.8 m in the main channel and 0.1 to 3.5 m on the floodplain. Physical characteristics and water quality varied substantially among pools with systematic differences between mesohabitats (main channel vs. floodplain pools) and moderate correlation among some environmental parameters (Supplementary Figs. S1, S2). All models reached convergence as indicated by all parameters with Gelman-Rubin statistics < 1.06. Model fidelity ranged between 69 and 97% among species with a total prediction accuracy of 84% (Supplementary Table S3), suggesting that the model is a useful description of the data-generating process. All model evaluation metrics and posterior parameter summaries are presented in the Supplement.

### Patterns in abundance

**Fig. 3:**
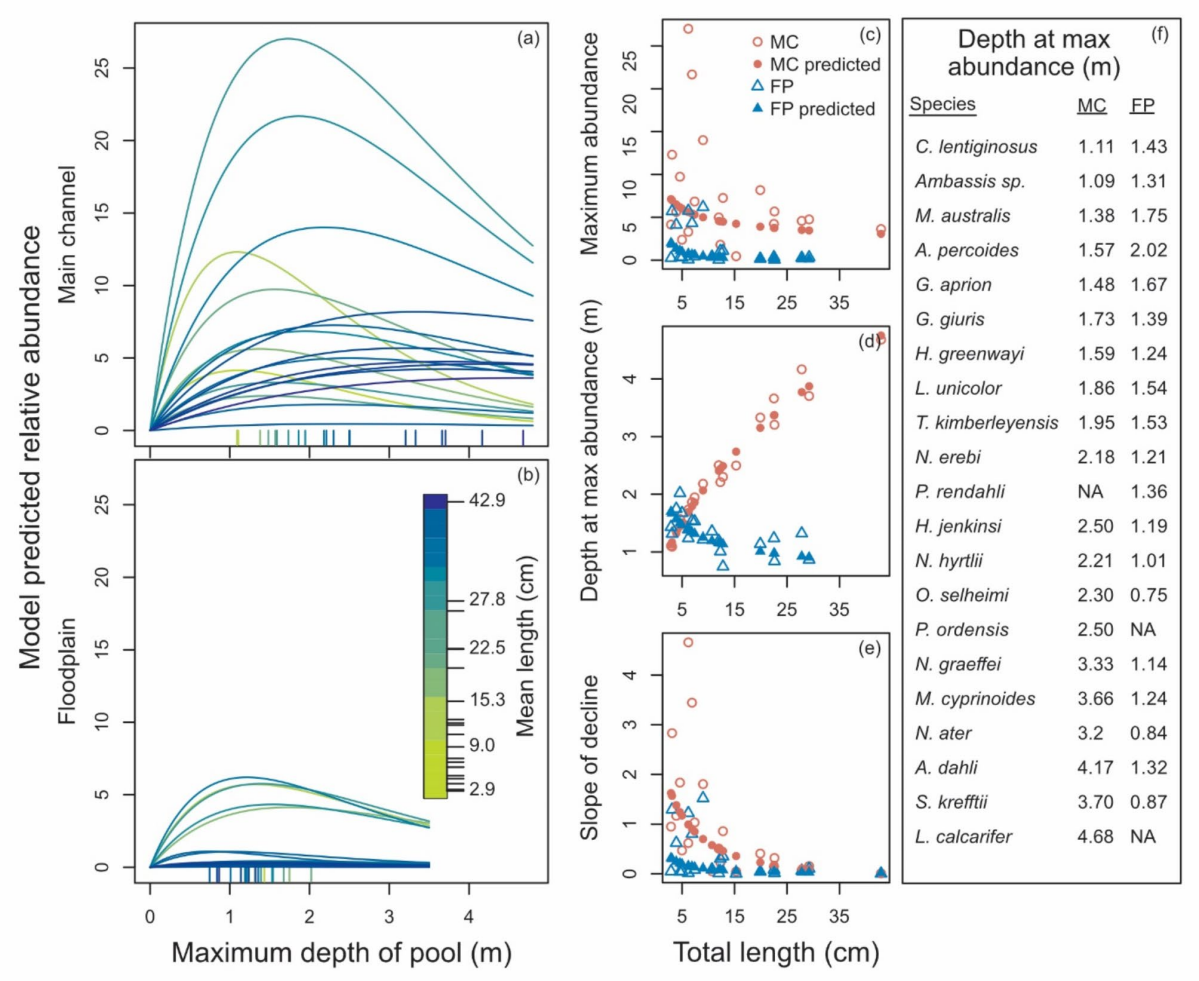
Species-level predicted abundance with maximum pool depth for main-channel and floodplain mesohabitats (**a**,**b**). (**c–e**) Depict the predicted pattern of species maximum abundance, depth at maximum abundances, and the slope of decline in abundance beyond the maximum, respectively. Open circles and triangles are predictions that account for among-species variation in model parameters beyond that explained by species length.

Our species-distribution model described large variation in abundance among fish species, mesohabitats, and all environmental variables. All fish species tended to have lower abundances in floodplain pools relative to main-channel pools (Fig. 3a–c). Fish abundances tended to increase with pool depth to a maximum and decline with additional increases in depth (Fig. 3a, b). The shape of this pattern was systematically related to the mean length of fish species as evidenced by a statistically significant length effect for ${\beta }_{1,i}$ and ${\beta }_{4,i}$ in Supplementary Table S4. The emergent patterns predicted higher maximum abundances for smaller species in both mesohabitats (Fig. 3c), but with smaller species strongly favouring shallower pools and larger species strongly favouring deeper pools only in the main channel (see red dots of Fig. 3d). Size-related patterns were subdued on the floodplain (Fig. 3c, d,e) but suggested higher abundance of smaller species in deeper pools and vice versa for larger species (see blue dots, Fig. 3d).

**Fig. 4:**
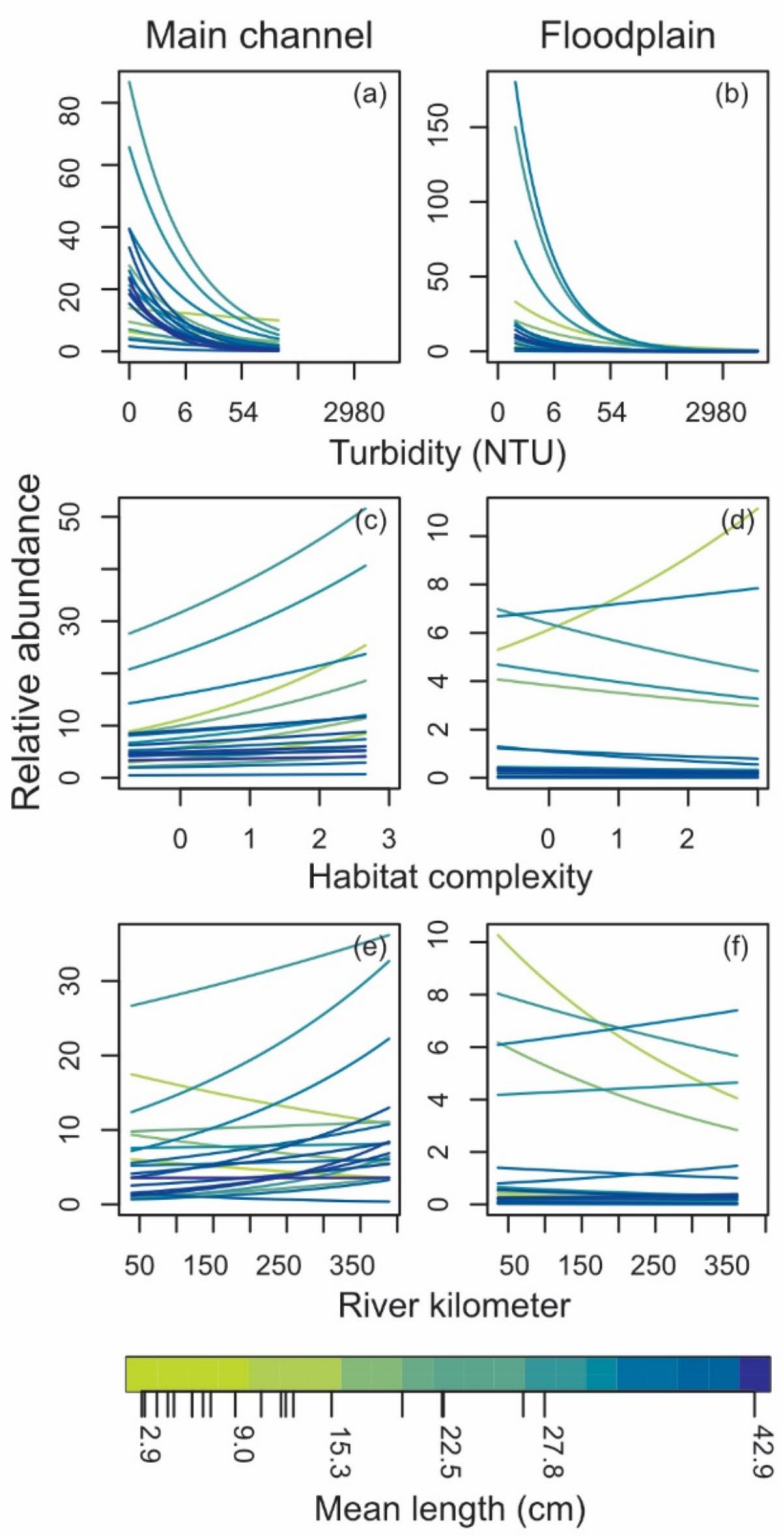
Species-level predicted abundance in main channel and floodplain pools across different water turbidity (NTU) (**a**,**b**), habitat complexity (**c**,** d**), and river kilometer (**e**,** f**). Note, river km refers to the distance upstream of the estuary. Each line represents a separate species with colour indicating the mean length of the species caught during the study.

Fish abundance varied with water turbidity, structural complexity, and river kilometre with emergent patterns shown for fish species length, although length effects were not statistically significant (Supplementary Table S4). The effect of turbidity on abundance tended to be strongly negative for all species (Fig. 4a, b) with 14 species demonstrating statistically significant negative effects in the main channel (Supplementary Fig. S5). These negative effects tended to be magnified for floodplain pools with four species demonstrating statistically significant negative interactions between mesohabitat and turbidity (Supplementary Fig. S5). The model predicted stronger effects for larger species with abundance reduced by 80% for 15 fish species at a turbidity of 41 NTU and 23 NTU in the main channel and floodplain pools respectively, and for all species at a turbidity of 1752 NTU on the floodplain (Fig. 4a, b). Compared to turbidity, fish abundance was influenced less by structural complexity (see effect sizes of Supplementary Fig. S5). However, five small species demonstrated statistically significant positive relationships to structural complexity in the main channel (Fig. 4c, Supplementary Fig. S5). In floodplain pools the importance of structural complexity was muted as negative interactions with mesohabitat effectively cancelled the positive main effect for smaller species, three of which were statistically significant (Fig. 4d, Supplementary Fig. S5). This pattern suggests a greater benefit of structural habitat in main-channel pools compared to floodplain pools for smaller fish species. Main and interaction river-kilometre effects were statistically significant for six species suggesting variation in distribution relative to the river estuary with little interaction with species length (Fig. 4e, f, Supplementary Fig. S5).

### Patterns in catchability

**Fig. 5:**
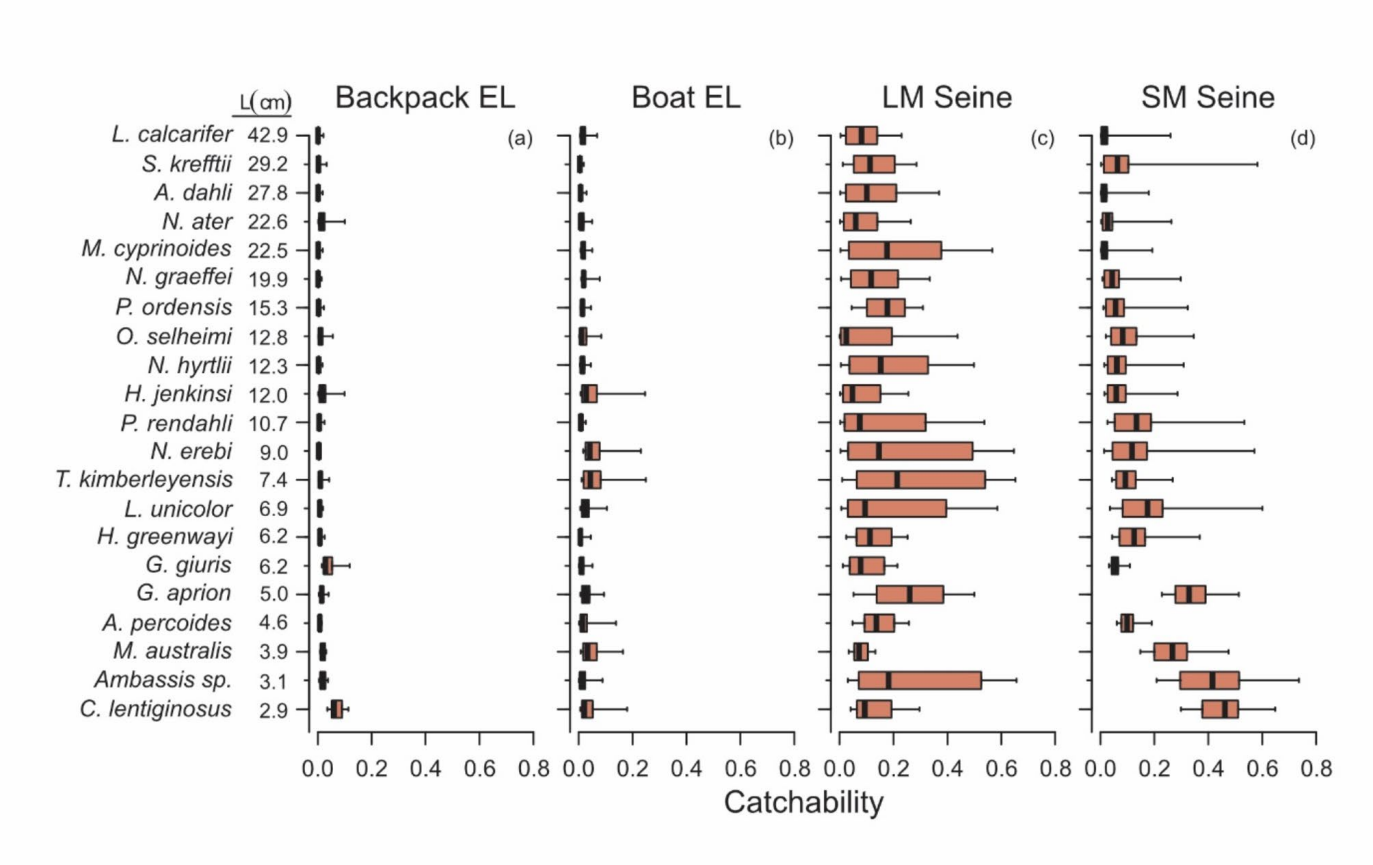
Boxplots showing variation in catchability (as a proportion) among samples collected with each sampling methods. Whiskers indicate the 90th percentiles (lower 5% and upper 95%). *EL* electrofishing, *LM* large mesh, *SM* small mesh.

Catchability was highly variable for all sampling methods and fish species. The model predicted that electrofishing methods had the lowest catchabilities for our study, with a mean of 0.03 and 0.06 for backpack and boat electrofishing, respectively (Fig. 5a, b). By contrast seines produced mean catchabilities of 0.21 and 0.15 for large-mesh and small-mesh seines respectively (Fig. 5c, d). Seine-based capture methods also demonstrated greater variation in catchability among pools, sample replicates and species (Fig. 5b, c).

**Fig. 6:**
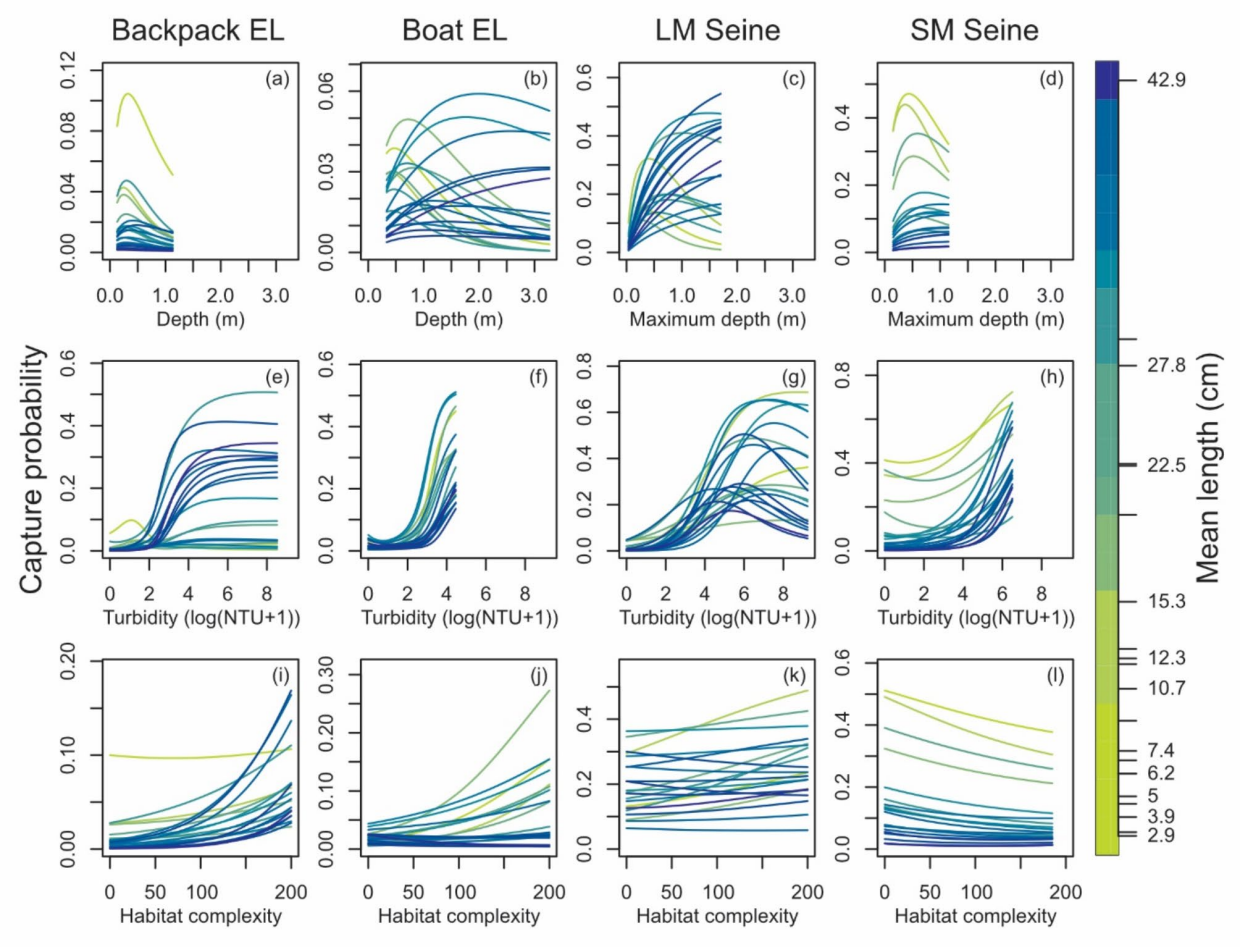
Relationship of capture probability to water depth, turbidity, and habitat complexity at sample locations. Plotted for four sampling methods and each fish species observed in the Fitzroy River fish assemblage. *Backpack EL* backpack electrofishing, *Boat EL* boat electrofishing, *LM Seine* large-mesh seine, *SM Seine* small-mesh seine. Note, the y axis varies among sampling gears and environmental covariates.

Environmental characteristics strongly influenced fish catchability. The relationship between catchability and depth was complex and varied with sampling gear and species with systematic relationships to species’ mean length (Fig. 6a-d). Fish length moderated the effect of depth on catchability with statistically significant length-depth interaction effects for all sampling methods (Supplementary Table S5). In general, smaller species tended to have the highest catchabilities in shallow-water samples with all methods while larger species tended to have the highest catchabilities in deep-water samples of for all methods (Fig. 6). These results were most precise for boat electrofishing and large-mesh seines (Supplementary Figs. S7, S8), and highly uncertain for backpack electrofishing (Supplementary Fig. S6), likely due to lower mean catchabilities and less samples to support analysis. Catchability generally increased with turbidity for most species and most sampling methods and revealed an optimum for large-mesh seines (Fig. 8, Supplementary Figs. S6–S9). For small-mesh seines, the turbidity effect interacted with fish size such that the catchability of larger species was affect more by turbidity (Fig. 6h, Supplementary Table S5). Complex structure primarily influenced catchability for electrofishing sampling methods (Fig. 6i, j, Supplementary Figs. S6, S7). For backpack electrofishing, this effect was statistically related to length (Supplementary Table S5), with increasing catchability with increasing complex structure for larger species (Supplementary Fig. S6). Boat electrofishing demonstrated 10 species with statistically significant effects of complex structure (Supplementary Fig. S7) that had no relationship to fish length.

### Management scenarios

**Fig. 7:**
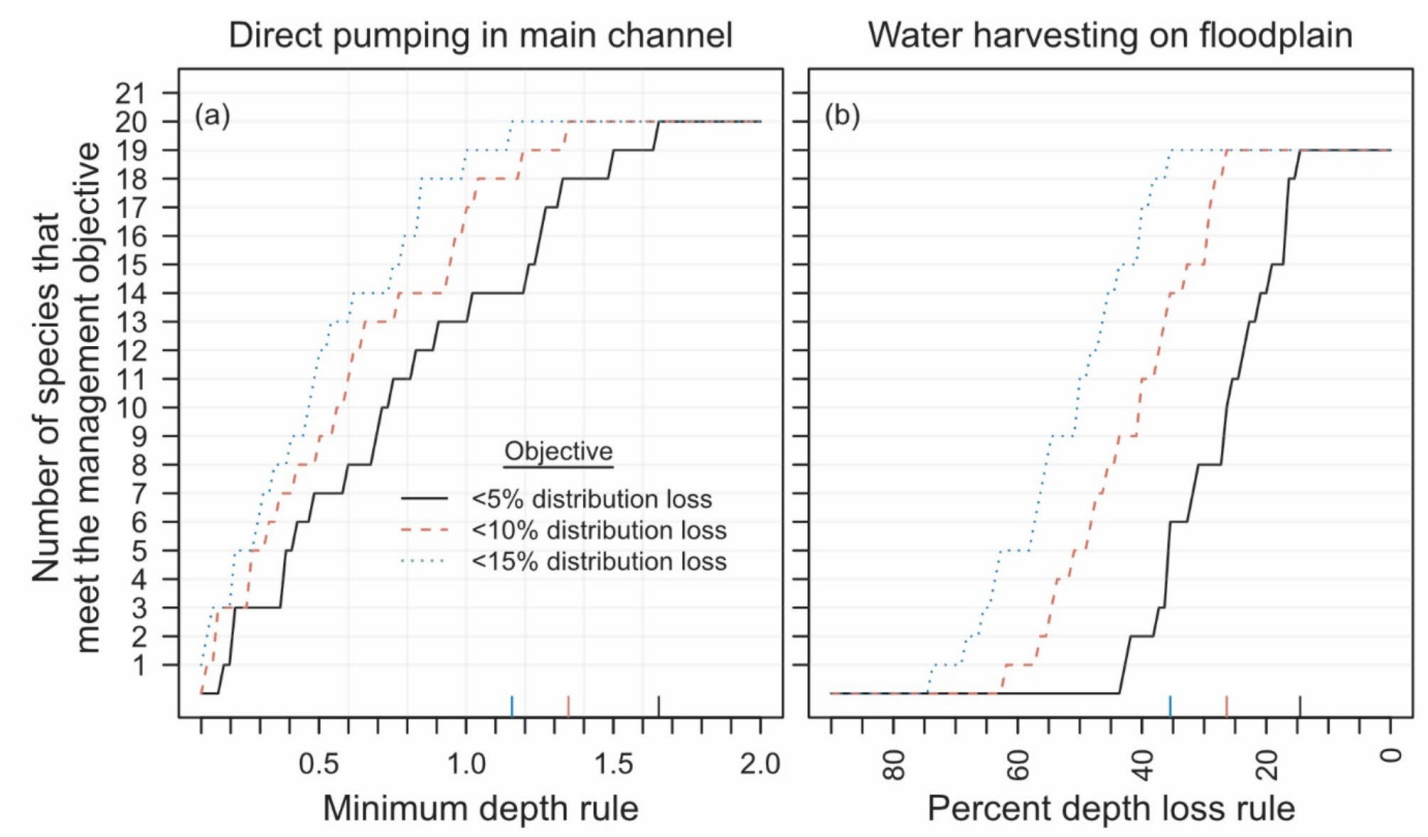
The number of fish species that meet the management objective of < 5%, < 10% and < 15% loss in distribution (i.e., the number of pools occupied) for (**a**) different minimum depth rules for pumping water directly from main channel pools, and (**b**) different percent reductions in the depth of floodplain pools associated with floodplain water harvesting. The solid colored lines that bisect the x-axis indicate the location where the three management objectives start to decline. Note, that the total number of species differs slightly between the two habitats.

Our management scenarios highlighted the tradeoff between increased water take and reduced distribution of fish in pools during the dry season and illustrated how water take rules could be established to meet different management objectives. For instance, our results suggest that meeting a conservative management objective of no more than 5% reduction of species distributions for the entire fish assemblage would require that water take via direct pumping in main-channel pools cease before maximum pool depths fall below a target of 1.65 m (Fig. 7a black line). This target could be relaxed to 1.35 m–1.25 m if management objectives accept 10% or 15% reduction in species distributions (Fig. 7a). Similarly, our results suggested that floodplain harvesting resulting in ≤ 14% reduction in maximum depths of floodplain pools was required to achieve a management objective of < 5% reduction in species distributions (Fig. 7b). However, more liberal management objectives that allow for 10% or 15% reduction in species distributions could be met with water harvesting targets that result in ≤ 26% or ≤ 36% reductions in maximum pool depths, respectively (Fig. 7b).

**Fig. 8:**
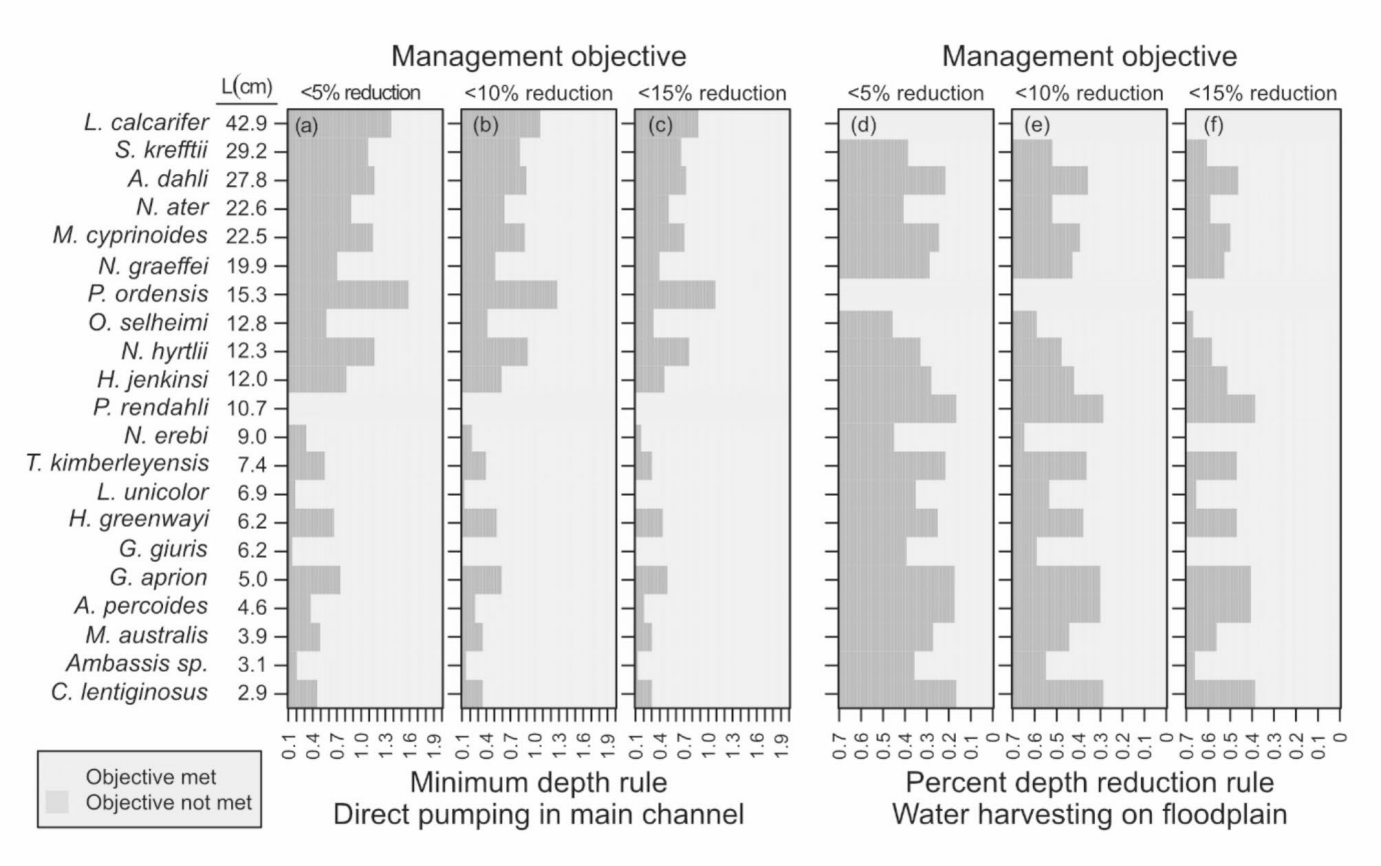
The take scenarios required to protect the distribution of each fish species under three management objectives which are < 5% loss in the distribution of the species (i.e., water take causes the species to be lost in < 5% of the pools where it would normally occur), < 10% loss in distribution, and < 15% loss in distribution. (**a–c**) Show the maximum depths required to protect fish from direct pumping in the main channel, and (**d–f**) show the percent of water take required to protect fish on the floodplain from floodplain harvesting (**d–f**).

Our evaluation of management scenarios highlighted fish species of potential vulnerability and illustrated how species-specific conservation objectives could be applied. For example, as lower minimum depth rules are considered for main-channel pools, the largest-bodied species *Planiliza ordensis*, and *Lates calcarifer* were predicted to be the first species that failed to meet the management objectives of < 5%, < 10%, and < 15% reduction in distribution. This suggests these species are vulnerable to water extraction from main-channel pools and may require the largest minimum depth rules to conserve (Fig. 8a–c). Alternatively, the small-bodied species *Glossogobius giuris*, *Leiopotherapon unicolor*, and *Ambassis sp*. were predicted to meet the management objectives for even the most liberal water take rules evaluated for main-channel pools, suggesting these species are robust to realistic scenarios of water extraction (Fig. 8a–c). The most vulnerable species to direct pumping of main-channel pools predicted by our analysis (*P. ordensis*, and *L. calicarifer*) do not typically utilize floodplain habitats and were, thus, excluded from the floodplain harvesting evaluation. As a result, our analysis predicted the most sensitive species to flood plain harvesting to include the small-bodied species *Craterocephalus lentiginosus*, *Amniataba percoides*, *Glossamia aprion*, and *Porochilus rendahli* (Fig. 8d–f). Alternatively, the most robust species to floodplain harvesting spanned a wide range of sizes and included *Nematalosa erebi*, *Oxyeleotris selheimi*, *G. giuris*, *L. unicolor*, and *Ambassis sp* (Fig. 8d–f).

## Discussion

Our model identified species-specific relationships between fish abundance/occurrence and pool depth. Relationships with depth demonstrated systematic variation with fish size, mesohabitat type (main channel, floodplain), and other environmental characteristics. These patterns indicate that water extraction will likely result in disproportionate reductions to the distribution of larger fish species in the Fitzroy River. Similar relationship among capture probability, depth, and fish size were consistently found for all sampling methods, highlighting the importance of considering the sampling process when evaluating fish depth preferences. The consistent link between fish-depth relationships and fish size suggest that our results may be generalizable to other data-deficient systems in the region.

A large body of literature exists on the role of temporarily isolated water bodies in intermittent river systems and the relationship of fish assemblage structure to hydro-morphological characteristics, including water depth see^49^ for a review. Many studies have taken place in Australian intermittent rivers e.g.^22–24,50^, but variation in methodology and the absence of accounting for depth-related variation in capture probability make direct comparisons with the Fitzroy River difficult. However, this body of research suggests that aspects of, or related to, pool depth are powerful determinants of fish survival and assemblage structure and operate through mechanisms including competition, predation, and water chemistry. For example, Keller^51^ determined patterns in fish abundance with water depth in isolated components of the Daly River of northern Australia. Eight of the fish species examined by Keller^51^ are included in the present study (e.g., *L. calcarifer*, *N. erebi*, *P. ordensis*) and demonstrate analogous patterns in abundance relative to water depth see Fig. 2 in Ref.^51^. The functional forms of the depth relationships are remarkably similar to those estimated for the Fitzroy River and follow similar patterns with fish size; however, their results suggest that these species favour shallower depths in the Daly River. This difference could be due to inherent differences in interacting biotic and abiotic factors between these river systems, as the Daly River exhibits less seasonal intermittency than the Fitzroy River. Furthermore, differences in sampling and analysis methodology would be expected to result in variation between results. Regardless of the cause of discrepancy, the similarity in patterns and the close link of these patterns to species size suggest some level of transferability among systems that may be used to set management rules in instances where data are lacking.

We found that patterns in fish distributions, at the pool and mesohabitat scale were explained well by the size of fish species. Habitat partitioning of fish across sizes is well corroborated in the ecological literature. The divergent association of large and small fish with deeper and shallower habitats has been demonstrated in systems across Europe^26,43^, North America^28,52^, South America^27^, and Australia^51,53^ including the Fitzroy River^50^. The association of larger fish with deeper habitats, such as *L. calcarifer*, *Strongylura krefftii*, and *Megalops cyprinoides*, is hypothesized to be a function of the broader range of niche availability to support resource needs for species of higher trophic status^26^. The concurrent partitioning of smaller fish into shallower habitats, such as *Ambassis sp.*, *C. lentiginosus*, and *Melanotaenia australis*, is often attributed to reduced predation risks associated with less large predatory species^54^. We observed this pattern in the main channel of the Fitzroy River with smaller species favouring shallower pools. However, this pattern did not exist on the floodplain where there were few large-bodied species to drive potential predator-driven partitioning.

In addition to depth associations, fish size is known to be closely linked to life-history attributes that determine population productivity. Fish size has been shown to strongly correlate with natural mortality rates, generation time, age at maturation, metabolism, and fecundity^55,56^. These life-history invariants have been described for over 1000 fish species by Pauly^57^ and over 30,000 fish species by Thorson^58^, demonstrating extraordinary consistency across marine and freshwater environments, globally. The general patterns that emerge from this literature conform to established life-history theory for fish^56,59^ with smaller fish tending towards opportunistic life-history strategies with short generation time and early maturation, and larger fish tending towards periodic life-history strategies with longer generation time and late maturation. These characteristics are known drivers of recolonization potential, important in intermittent rivers that rely on isolated refuge pools for seasonal repopulation of previously dry riverbeds and explosive reproductive outputs. The fish species included in this study demonstrate variation in life-history traits along this established continuum^60^ suggesting that larger species of the Fitzroy River, such as *L. calcarifer* (barramundi) and *P. pristis* (freshwater sawfish) will be particularly vulnerable to local extirpations due to systematic reductions in pool depth.

Our evaluation of conservation outcomes of water extraction via main-channel pumping and floodplain harvesting provides stylized examples of how information can be synthesized for management. We developed these regulatory scenarios in cooperation with water managers to represent rules that could be applied to water licences. The evaluation suggested that larger-bodied fish species may be the most vulnerable to water extraction. Furthermore, the evaluation suggested that minimum water depth rules > 1.65 m for main-channel pools and < 14% reduction of mean pool depths for floodplain pools as potential guidelines for the conservation of the natural fish assemblage structure. However, an important limitation to consider when interpreting these conclusions is our use of space-for-time substitution in our experimental design. Specifically, we sampled pools of varying depths and various states of drying across the landscape as opposed to sampling pools with depths being reduced due to drying through time. Thus, our policy predictions require the assumption that similar processes and rates determine patterns in fish assemblage structure related to depth changes through time as through space. Although, this assumption will be violated to an unknown degree, we expect that our conclusions are acceptably robust for guiding early policy development for two main reasons. Firstly, processes such as competition, predation, and water quality will strongly influence the distribution of species across the landscape of isolated pools as well as through time within isolated pools; and, secondly, our results corroborate patterns in assemblage structure relative to fish species size, richness, abundance, and water depth described by a large body of international literature on isolated drying pools^61–65^ and connected pools^28,51,66^ with marked agreement. That these patterns demonstrate consistency across systems suggests that our findings are broadly representative and provide reasonable targets for initial precautionary policy development. Further research on the impacts of water extraction on the assemblage structure and distribution of fish of intermittent rivers via adaptive management experimentation, could provide strong causal links and represent a valuable contribution to the literature.

We structured this analysis based on relevance to management and biological realism to promote efficient use of data and future sharing of information within and among systems. One key choice was including the interactions between fish length and all environmental covariates. Investigating species-environmental interactions has been discussed in the ecological literature and termed the ‘fourth corner solution’^33^. Fourth corner solution postulates that the interactions between species and environmental characteristics determine the distribution of species assemblages. Our novel application of analogous interactions in our observation sub-models extend this idea by postulating that interactions between sampling, species, and environmental characteristics determine variation in capture probability of species e.g^67^. There are several benefits of these model structures that include increased model resolutions due to reductions in model dimensionality and increased transferability due to more accurately describing mechanistic relationships that are conserved across study systems. The emergence of fish length as a good predictor of environmental covariate effects and the strong alignment of our model results, regarding species distributional and sampling characteristics, with a large body of international research, suggests that they may be generalizable. Formal testing to validate the transferability of these patterns could be achieved by applying analogous models to rivers with existing similarly-collected data, such as the Daly River^51^. This exercise could establish model transferability across northern Australia and be used to generate depth relationships/rules to guide water development proposed for these rivers.

A similar process could be undertaken to validate and refine predictions of fish capture probability. Ongoing learning and adaptive management processes that support the refinement of management prescriptions require some form of periodic monitoring feedback on system states^68^. Our analysis described relationships of fish capture probability to sampling methods, fish length, depth, and turbidity that are well supported in the literature but have never been compiled into a single analysis as we have done here. This is particularly true regarding three-way interactions between sampling, species, and environmental characteristics see^67^ for an exception. Our comprehensive description of fish sampling properties provides an opportunity to create transferable models of fish capture probability that can be broadly applied through formal methods of information sharing such as Bayesian priors and data standardizations e.g^69^. Many attempts have been made to create generalizable models of fish capture probability but application beyond a single dataset has not been put into practice to our knowledge. Thus, capitalizing on these results as well as related research would make valuable use of this rich body of literature.

## Electronic supplementary material

Below is the link to the electronic supplementary material.

Supplementary Material 1

## Data Availability

Data are available in the UWA digital repository at https://research-repository.uwa.edu.au/en/datasets/.
