# Supplementary material for: Defining depth requirements to conserve fish assemblages from water take in an intermittent river
Source: Sci Rep. 2024 Dec 2;14:29863. doi: 10.1038/s41598-024-81339-5 (PMC11612426; doi:10.1038/s41598-024-81339-5)
Supplement: Supplementary file 1 — Supplementary Material 1 [file 41598_2024_81339_MOESM1_ESM.docx]

**SUPPLEMENTARY INFORMATION**

**Title**

Defining depth requirements to conserve fish assemblages from water take in an intermittent river

**Authors**

Daniel C. Gwinn, Leah S. Beesley, Bradley J. Pusey, Michael M. Douglas, Chris S. Keogh, Oliver Pratt, Tom Ryan, Mark J. Kennard, Thiaggo C. Tayer, Caroline A. Canham, Lewis G. Coggins Jr and Samantha A. Setterfield

**Fish caught**

Table S1. Fish species included in analysis, mean length caught, and maximum length of species.

| Species | Abr. | $\bar{L}$ | $L_{max}$ |
| --- | --- | --- | --- |
| *Craterocephalus lentiginosus* | *C. len* | 2.9 | 8 |
| *Ambassis sp.* | *Amb* | 3.1 | 5.5 |
| *Melanotaenia australis* | *M. aus* | 3.9 | 11 |
| *Amniataba percoides* | *A. per* | 4.6 | 20 |
| *Glossamia aprion* | *G. apr* | 5.0 | 25 |
| *Glossogobius giuris* | *G. giu* | 6.2 | 25 |
| *Hannia greenwayi* | *H. gre* | 6.2 | 14 |
| *Leiopotherapon unicolor* | *L. uni* | 6.9 | 33 |
| *Toxotes kimberleyensis* | *T. kim* | 7.4 | 18 |
| *Nematalosa erebi* | *N. ere* | 9.0 | 47 |
| *Porochilus rendahli* | *P. ren* | 10.7 | 24 |
| *Hephaestus jenkinsi* | *H. jen* | 12.0 | 40 |
| *Neosilurus hyrtlii* | *N. hyr* | 12.3 | 50 |
| *Oxyeleotris selheimi* | *O. sel* | 12.8 | 55 |
| *Planiliza ordensis* | *P. ord* | 15.3 | 60 |
| *Neoarius graeffei* | *N. gra* | 19.9 | 60 |
| *Megalops cyprinoides* | *M. cyp* | 22.5 | 130 |
| *Neosilurus ater* | *N. ate* | 22.6 | 75 |
| *Anodontiglanis dahli* | *A. dah* | 27.8 | 49 |
| *Strongylura krefftii* | *S. kre* | 29.2 | 85 |
| *Lates calcarifer* | *L. cal* | 42.9 | 180 |

Symbol definitions: $\bar{L}$ = mean standard length (cm) of all individuals measured; $L_{max}$ = maximum length (cm) of the species

**Sampling methodology**

Boat electrofishing was used in pools typically > 1-m deep and was performed by two operators (driver, netter). The boat (aluminium 4.4-m long x 1.8-m wide) was equipped with a 7.5 Kva HC80 electro-fishing unit (Mid-West Lakes), operated at 180-750 volts DC, 120 pulses sec^-1^, 25% duty cycle and 10-30 amps based on water conductivity. Samples targeted homogenous habitats (depth, structure) for 29-295 sec of power. A Smithroot LR20 backpack electrofishing fishing unit was used predominantly in shallow, clear-water habitats. The unit was operated at approximately 300-volts DC (120 pulses sec^-1^, 25% duty cycle and 1-3 amps), moving upstream. Samples targeted homogenous habitat for 17-173 sec of power. A 10-m long or a 5-m long beach seine was used depending on pool size and complex structure. Both seines had a 1-m drop with 9-mm mesh. Typically, the nets were dragged through the littoral zone perpendicular to the bank. A 7-m long beach seine with 1-m drop and 2-mm mesh was used to target smaller fish. The total number of samples collected per site visit ranged between two and 18. Sampling effort was recorded for electrofishing as seconds of power, and for seines as area of haul.

We recorded environmental variables at two spatial scales to represent characteristics of pools that may influence species distributions, and characteristics of sample locations that may influence species capture probability. At each pool, we recorded water quality parameters including dissolved oxygen (mg L^-1^), electrical conductivity (μS L^-1^), turbidity (NTU) and pH (Table 1). These were measured at a representative location using a Hydrolab Quanta (Hydrolab Corporation, Loveland, CO, U.S.A.) held 15-cm below the water surface prior to fish sampling. We recorded maximum depth, mean depth, average wetted channel width, and pool length. Mean depth was estimated using point measurements taken along cross-sectional transects (n = 2 - 5). Measurements were taken at 1-m or 5-m intervals along each transect in accordance with pool size and heterogeneity. Maximum depth was determined by a targeted survey to seek the deepest point. A laser rangefinder (Athlon 1200Y) was used to measure wetted width at each transect and total pool length. Length was capped at 2 km for very large pools. Percent composition of substrate type (sand, mud, gravel, cobble, rocks) and structural complexity (woody debris, rocks, roots, macrophytes, undercut banks) were approximated by visual survey for each sample replicate. Percent complex structure (structural/habitat complexity) was also determined at the site scale as part of the depth transects. At each 1-m or 5-m interval we estimated the percent cover of large and small woody debris, roots, undercut banks, mud boulders, and macrophyte beds for a 1m^2^ area. These values were summed for a summary value.

**Correlations among environmental variables**


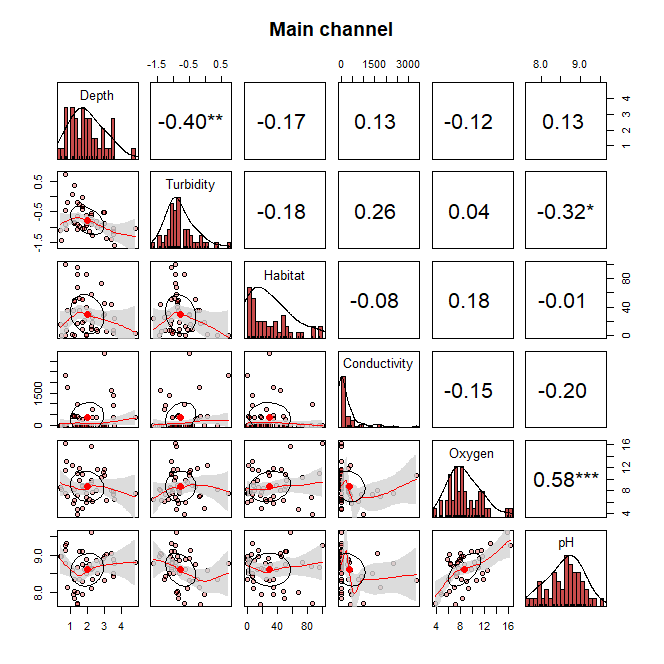


Figure S1. Correlation among environmental variables measured in pools sampled in the main channel of the Fitzroy River.


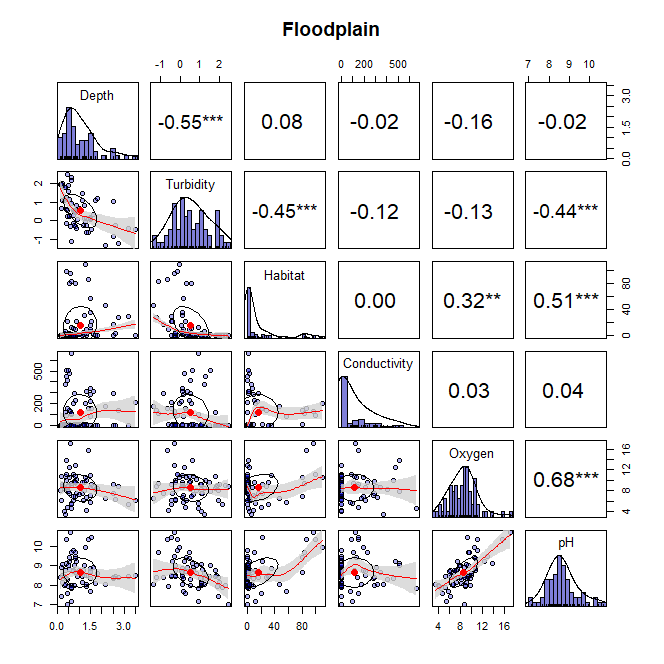


Figure S2. Correlation among environmental variables measured in pools sampled in the Floodplain of the Fitzroy River.

**Model code, evaluation and parameter summaries**

The first part of this document details the specification and code used to estimate fish relative abundance and relationships to covariates. The second part assesses model fit, and the third part provides parameter summaries generated from posterior probabilities.

**Model specification**

Our model is based on the hierarchical model first presented by Royle and Nichols (2003) that estimates a single species occurrence and abundance at multiple sites from repeated presence-absence data. The model was originally developed to account for heterogeneity in detection probability in occupancy models due to unobservable variation in abundance; however, by doing so, abundance becomes an estimable parameter. Like contemporary occupancy models and state-space models, this abundance model is made up of a sub-model describing patterns in abundance and a sub-model describing the observation process. The model of Royle and Nichols (2003) was later extended to a multispecies format by Yamaura et al. (2011) by connecting an ensemble of single species models with all parameters specified as random effects across species with community-level means. Here we further extend the multi-species version of the model to include multiple observation sub-models that integrate data from multiple sampling methods to capitalize on the unique performance of each sampling method for different fish species and environmental characteristics. Furthermore, we extend the model to describe species-specific parameters as a function of both random effects and fixed effects of species size.

We drew inference from 20,000 posterior samples resulting from four MCMC chains of 10^6^ iterations with the first 5^6^ discarded and remaining thinned to every 50^th^ value. Convergence of MCMC chains was diagnosed visually and with Gelman-Rubin statistics, with values ≤ 1.1 indicating adequate convergence (Gelman and Hill 2006). Posterior probability distributions of model parameters were estimated using a Gibbs sampler implemented in JAGS (Plummer 2003) called from within program R (R Development Core Team 2022) with the library R2jags (<http://mcmc-jags.sourceforge.net>). The JAGS code specifying the model is:

model{

###---OBSERVATION SUBMODELS---###

for(j in 1:nsp){

#BACKPACK ELECTROFISHING

for(i in 1:BPn){

yBP[j,i] ~ dbern(pBP[j,i])

pBP[j,i] <- 1-(1-rBP[j,i])^N[j,siteBP[i]]

rBP[j,i] <- (EBP[i]^eff[1,j])*(1-exp(-exp(qBP[j,i])))

qBP[j,i] <- BP[1,j] + log(2*depthBP[i]) - exp(BP[2,j])*2*depthBP[i] + BP[3,j]*turbBP[i] + BP[4,j]*pow(turbBP[i],2) + BP[5,j]*habBP[i] +

BP[6,j]*condBP [i]}

#BOAT ELECTROFISHING

for(i in 1:BEn){

yBE[j,i] ~ dbern(pBE[j,i])

pBE[j,i] <- 1-(1-rBE[j,i])^N[j,siteBE[i]]

rBE[j,i] <- (EBE[i]^eff[2,j])*(1-exp(-exp(qBE[j,i])))

qBE[j,i] <- BE[1,j] + log(2*depthBE[i]) - exp(BE[2,j])*2*depthBE[i] + BE[3,j]*turbBE[i] + BE[4,j]*pow(turbBE[i],2) + BE[5,j]*habBE [i] +

BE[6,j]*condBE [i]}

#SEINE (large mesh 10m and 5m)

for(i in 1: LMn){

yLM[j,i] ~ dbern(pLM[j,i])

pLM[j,i] <- 1-(1-rLM[j,i])^N[j,siteLM[i]]

rLM[j,i] <- (ELM [i]^eff[3,j])*((s5ind[i]*reduce)+(1-s5ind[i]))*(1-exp(-exp(qLM[j,i])))

qLM[j,i] <- LM[1,j] + log(2*depthLM[i]) - exp(LM[2,j])*2*depthLM[i] + LM [3,j]*turbLM[i] + LM[4,j]*pow(turbLM[i],2) + LM[5,j]*habLM[i]}

#SEINE (fine mesh 7m)

for(i in 1:SMn){

ySM[j,i] ~ dbern(pSM[j,i])

pSM[j,i] <- 1-(1-rSM[j,i])^N[j,siteSM[i]]

rSM[j,i] <- (ESM[i]^eff[4,j])*(1-exp(-exp(qSM[j,i])))

qSM[j,i] <- SM[1,j] + log(2*depthSM[i]) - exp(SM[2,j])*2*depthSM[i] + S7[3,j]*turbSM[i] + S7[4,j]*pow(turbSM[i],2) + SM[5,j]*habSM[i]}

###---ABUNDANCE SUBMODEL---###

for(i in 1:nsttr){

N[j,i] ~ dpois(lam[j,i])

lam[j,i] <- exp(lamlog[j,i] + epi1[j,site[i]] + epi2[j,i])

lamlog[j,i] <- bet[1,j] + bet[3,j]*off[i] + log(Dmax[i]) - exp(bet[2,j]+ bet[4,j]*off[i])*Dmax[i] + bet[5,j]*turb[i] + bet[6,j]*turb[i]*off[i]

+ bet[7,j]*habcom[i] + bet[8,j]*habcom[i]*off[i] + bet[9,j]*rkm[i] + bet[10,j]*rkm[i]*off[i]

###---HYPERDISTRIBUTIONS---###

##--abundance submodel

epi2[j,i] ~ dnorm(0,tau[1])

}#i

for(i in 1:nst){epi1[j,i] ~ dnorm(0,tau[2])}

for(i in 1:10){bet[i,j] ~ dnorm(betmu[i,j],tau[i+2])}

##--observation submodels

for(i in 1:6){BP[i,j] ~ dnorm(bp[i,j],tau[i+12])}

for(i in 1:7){BE[i,j] ~ dnorm(be[i,j],tau[i+18])}

for(i in 1:6){LM[i,j] ~ dnorm(lm[i,j],tau[i+25])}

for(i in 1:6){SM[i,j] ~ dnorm(sm[i,j],tau[i+31])}

###---HYPER-PARAMETERS DESCRIBED BY FISH LENGTH---###

for(i in 1:10){ betmu[i,j] <- eta[i] + eta[i+10]*ltl[j]}

for(i in 1:6){bp[i,j] <- bp.phi[i] + bp.phi[i+6]*ltl[j]}

for(i in 1:7){be[i,j] <- be.phi[i] + be.phi[i+7]*ltl[j]}

for(i in 1:6){lm[i,j] <- lm.phi[i] + lm.phi[i+6]*ltl[j]}

for(i in 1:6){sm[i,j] <- sm.phi[i] + sm.phi[i+6]*ltl[j]}

}#j

###---PRIORS---###

eta[1]~dnorm(1,.04)

eta[2]~dnorm(1,.04)

eta[3]~dnorm(0,.04)

eta[4]~dnorm(0,.04)

for(i in 5:20){eta[i] ~ dnorm(0,.1)}

for(i in 1:2){

bp.phi[i] ~ dnorm(0,.04)

be.phi[i] ~ dnorm(0,.04)

lm.phi[i] ~ dnorm(0,.04)

sm.phi[i] ~ dnorm(0,.04)}

for(i in 9:50){phi[i] ~ dnorm(0,.1)}

for(i in 1:4){eff[i] ~ dbeta(1.1,1.1)}

reduce ~ dbeta(1.1,1.1)

for(i in 1:37){tau[i] <- pow(sig[i],-2)

sig[i] ~ dt(0,2/(2^2),2)T(0,5)}

###---REGULARIZATION---###

penalty <- (sum(abs(eta[5:20]))+sum(abs(bp.phi[3:6]))+ sum(abs(be.phi[3:7]))+ sum(abs(lm.phi[3:6]))+ sum(abs(sm.phi[3:6]))+

sum(abs(BP[3:6,]))+sum(abs(BE[3:7,]))+ sum(abs(LM[3:6,]))+sum(abs(SM[3:6,]))+sum(abs(bet[5:10,])))/100

pen ~ dt(0,2/(.1^2),2)T(0,3)

pentau <- pow(pen,-2)

zero ~ dnorm(penalty,pentau)

}

In the preceding JAGS model code, the abbreviations BP, BE, LM, and SM are used to indicate backpack electrofishing, boat electrofishing, large-mesh 10m and 5m seines and fine-mesh 7m seines. All parameter definitions follow.

Data:

yBP, yBE, yLM, ySM – Indicates binary detection/non-detection data collected with each sampling method.

EBP, EBE, ELM, ESM – These are vectors of sampling effort which are scaled to range in values from 0-1.

depthBP, depthBE – the average depth of the electrofishing transect (scaled between zero and one by dividing by the maximum value).

depthLM, depthSM – the maximum depth of the seine haul (scaled between zero and one by dividing by the maximum value).

turbBP, turbBE, turbLM, turbSM – The turbidity value at the pool during sampling (i.e., turb value).

habBP, habBE – the habitat complexity score for the area of the electrofishing transect (centred on zero and scaled to one standard deviation).

habLM, habSM – the habitat complexity score for the area of the seine haul (centred on zero and scaled to one standard deviation).

condBP, condBE – the electrical conductivity of the pool during sampling (centred on zero and scaled to one standard deviation).

Dmax – the maximum depth of the pool during sampling (scaled between zero and one by dividing by the maximum value).

turb – the turbidity of the pool during the sampling event (log+1 transformed, centred on zero and scaled to one standard deviation)

habcom – the habitat complexity score of the pool during the sampling event (centred on zero and scaled to one standard deviation).

rkm – the distance of the pool from the estuary (centred on zero and scaled to one standard deviation).

off – a binary indicator of when a pool in located on the floodplain.

ltl – natural log of mean total length of each fish species (centred on zero and scaled to one standard deviation).

Data for indexing:

nsp – The number of species in the data set.

nsttr – The total number of combinations of pools and sampling events.

nst – The total number of pools.

BPn, BEn, LMn, SMn – The total number of samples collected by each sampling methods.

siteBP, siteBE, siteLM, siteSM – These are vectors that index the pool and sampling event where and when the sample was collected.

S5ind – A binary indicator of when the 5-m seine was used.

Derived quantities:

pBP, pBE, pLM, pSM – Indicates the probability of detecting at least one individual of each species for each sampled (i.e., detection probability).

rBP, rBE, rLM, rSM – Indicates the average probability of capture of individual fish for each species and sample (i.e., capture probability).

qBP, qBE, qLM, qSM – Indicates the average probability of capture of individual fish per unit of sampling effort for each species and sample (i.e., catchability).

lam – expected abundance for each species and pool during sampling.

lamlog – the natural log of expected abundance, lam.

Estimated parameters:

eff – effect of sampling effort on capture probability.

BP, BE, LM, SM – species-specific sample scale effects of environmental covariates on capture catchability.

reduce – parameter that scales the catchability of 5-m seines relative to 10-m seines.

N – the relative abundance of each fish species at each pool during each visit.

epi1 – random effect of pool on abundance of each fish species.

epi2 – random effect of site-visit on abundance of each fish species.

bet – species-specific effects of pool-scale environmental covariates on abundance.

eta – fixed effects of fish species average length on environmental covariate effects of abundance (bet).

bp.phi, be.phi, lm.phi, sm.phi, – fixed effects of fish species average length on environmental covariate effects of catchability.

**Prior distributions specifications**

We specified prior distributions wide enough to cover plausible effect sizes while avoiding large values when the data provide little information. Prior distributions are presented in Table S2 and plotted with posterior distributions in Figure S3.

Table S2 Prior distributions applied in model.

| Parameters | Prior distribution |
| --- | --- |
| bet[1:2] | Normal($\mu=1$,$\sigma=5$) |
| bet[3:20] | Normal($\mu=0$,$\sigma=3$) |
| bp.phi[1:2], be.phi[1:2], lm.phi[1:2],sm.phi[1:2] | Normal($\mu=0$,$\sigma=5$) |
| bp.phi[3:12],be.phi[3:12],  lm.phi[3:10],sm.phi[3:10] | Normal($\mu=0$,$\sigma=3$) |
| eff[1:4],reduce | Beta($\alpha=1.1$,$\beta=1.1$) |
| sig[1:34] | Student-t($\mu=0$,$scale=2$,$df=2$) |


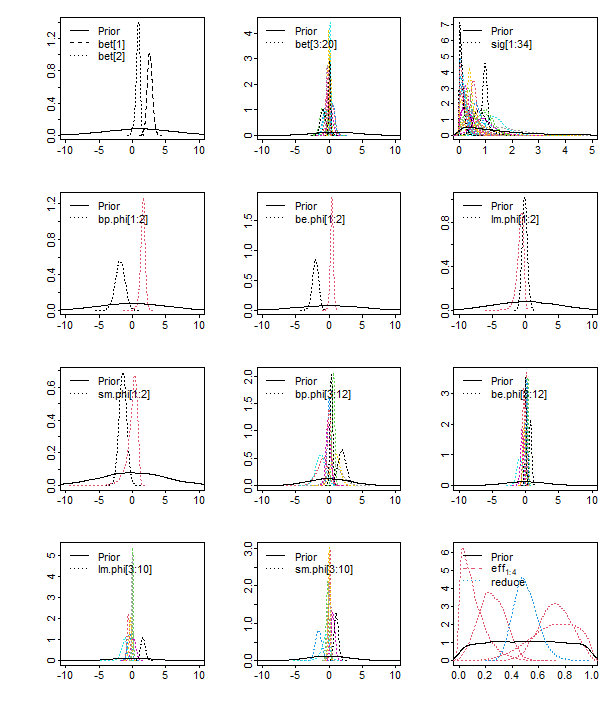


Figure S3. Prior distributions plotted with posterior distributions to demonstrate adequate width of priors.

**Regularization**

We performed regularization of our model for the purpose of moderating model complexity, reducing parameter uncertainty, improving predictive properties, and promoting mixing of MCMC chains. We achieve this regularization by including a penalty on the model likelihood that was proportional to the sum of the absolute values of a selection of nuisance parameters unrelated to our primary research question of water depth. The penalty was formulated as,

| $0\sim\mathrm{Normal}\left( \sum\left\vert\theta\right\vert,\sigma\right)$ | (1) |
| --- | --- |
| $\sigma\sim\mathrm{couchy}\left( 0,200,2 \right) T(0,2)$ | (2) |

where zero (0) in equation 1 is supplied as a fixed value and $\theta$ is the parameter set to be regularized. The standard deviation $\sigma$ in equation 1 controls the strength of the penalty with smaller values increasing the strength of shrinkage applied to the parameters contained in the set $\theta$. Because we do not know the optimal level of shrinkage, we treat the selection of $\sigma$ as an estimation problem (e.g. Leng et al. 2014), and specify its prior as a truncated half Cauchy (equation 2). This form of regularization is similar to Bayesian adaptive Lasso (Leng et al. 2014, Lu and Lou 2022) because unique shrinkage is applied to each model parameter relative to support in the data. We applied the regularization across multiple hierarchical layers by including all species-specific random effects and all fixed effects associated with environmental covariates in the abundance and observation sub-models. Parameters describing the relationships of abundance and catchability with depth as well as catchability with sampling effort do not simplify to plausible models when shrunk to zero; thus, parameters associated with these effects were not included in the $\theta$ parameter set.

**Model fit**

We assessed model fit by calculating the probability of each data point from the posterior predictive distribution. The average probability of correctly predicting the data are summarized by species and sampling methods in Table S3.

Table S3. The average probability of predicting the data correctly by the fitted model.

| Species | Backpack  electrofishing | Boat  electrofishing | Large-mesh  seine | Small-mesh  seine | Mean |
| --- | --- | --- | --- | --- | --- |
| *C. lentiginosus* | 0.75 | 0.92 | 0.87 | 0.76 | 0.86 |
| *Ambassis sp.* | 0.67 | 0.84 | 0.79 | 0.69 | 0.78 |
| *M. australis* | 0.73 | 0.76 | 0.77 | 0.59 | 0.74 |
| *A. percoides* | 0.77 | 0.77 | 0.77 | 0.93 | 0.79 |
| *G. aprion* | 0.87 | 0.89 | 0.85 | 0.87 | 0.86 |
| *G. giuris* | 0.58 | 0.66 | 0.72 | 0.73 | 0.69 |
| *H. greenwayi* | 0.90 | 0.93 | 0.89 | 0.97 | 0.92 |
| *L. unicolor* | 0.64 | 0.66 | 0.74 | 0.63 | 0.69 |
| *T. kimberleyensis* | 0.74 | 0.67 | 0.79 | 0.89 | 0.76 |
| *N. erebi* | 0.78 | 0.7 | 0.82 | 0.64 | 0.75 |
| *P. rendahli* | 0.98 | 0.99 | 0.93 | 0.91 | 0.95 |
| *H. jenkinsi* | 0.75 | 0.75 | 0.95 | 0.98 | 0.87 |
| *N. hyrtlii* | 0.96 | 0.90 | 0.85 | 0.90 | 0.88 |
| *O. selheimi* | 0.75 | 0.79 | 0.90 | 0.85 | 0.84 |
| *P. ordensis* | 0.94 | 0.98 | 0.96 | 1.00 | 0.97 |
| *N. graeffei* | 0.91 | 0.68 | 0.85 | 0.97 | 0.81 |
| *M. cyprinoides* | 0.93 | 0.81 | 0.88 | 0.99 | 0.87 |
| *N. ater* | 0.71 | 0.85 | 0.94 | 1.00 | 0.90 |
| *A. dahli* | 0.93 | 0.88 | 0.89 | 0.98 | 0.90 |
| *S. krefftii* | 0.90 | 0.93 | 0.85 | 0.95 | 0.90 |
| *L. calcarifer* | 0.91 | 0.84 | 0.96 | 1.00 | 0.92 |
| Mean | 0.81 | 0.82 | 0.86 | 0.87 | **0.84** |

**Parameter summaries generated from posterior probabilities**


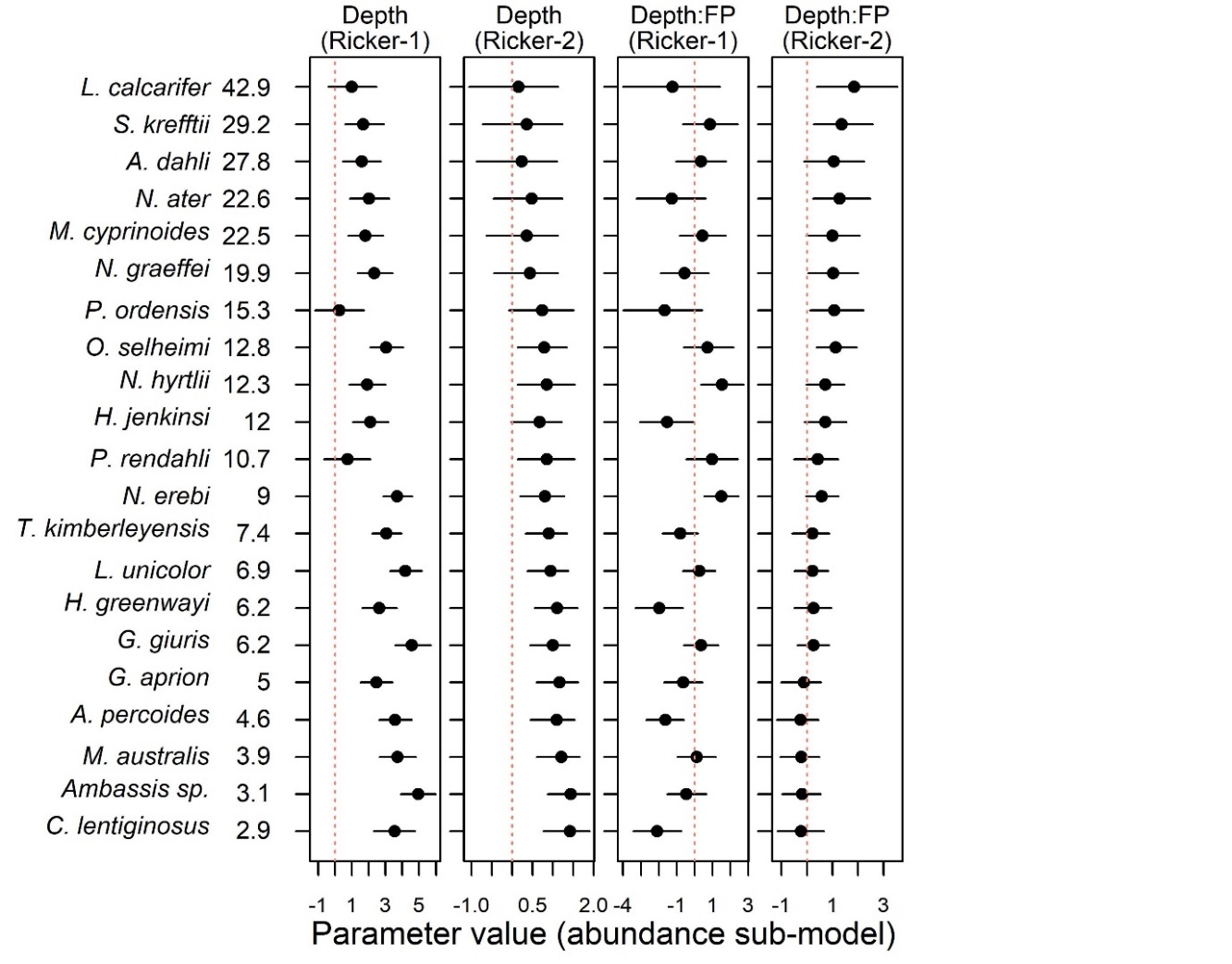


Figure S4. Parameter values for environmental covariates in the abundance sub-model (part one of two). The parameter Depth (Ricker-1) refers to the first depth parameter in Ricker model ($\beta_{1,i}$) for main channel and Depth (Ricker-2) refers to the second depth parameter in Ricker model ($\beta_{2,i}$) for the main channel. Depth:FP (Ricker-1) and (Ricker-2) refers to the same parameters for floodplain pools ($\beta_{3,i})$ and ($\beta_{4,i}$) respectively. Fish species are ordered from largest (top) to smallest (bottom) in accordance with their mean length (cm) collected during the study (length data to the right of their name). The black circular symbol indicates the mean and the whiskers the 90% Bayesian Credible Intervals. The dashed vertical red line indicates an effect of zero. Beta parameters are shown in equation 1 in the main text.


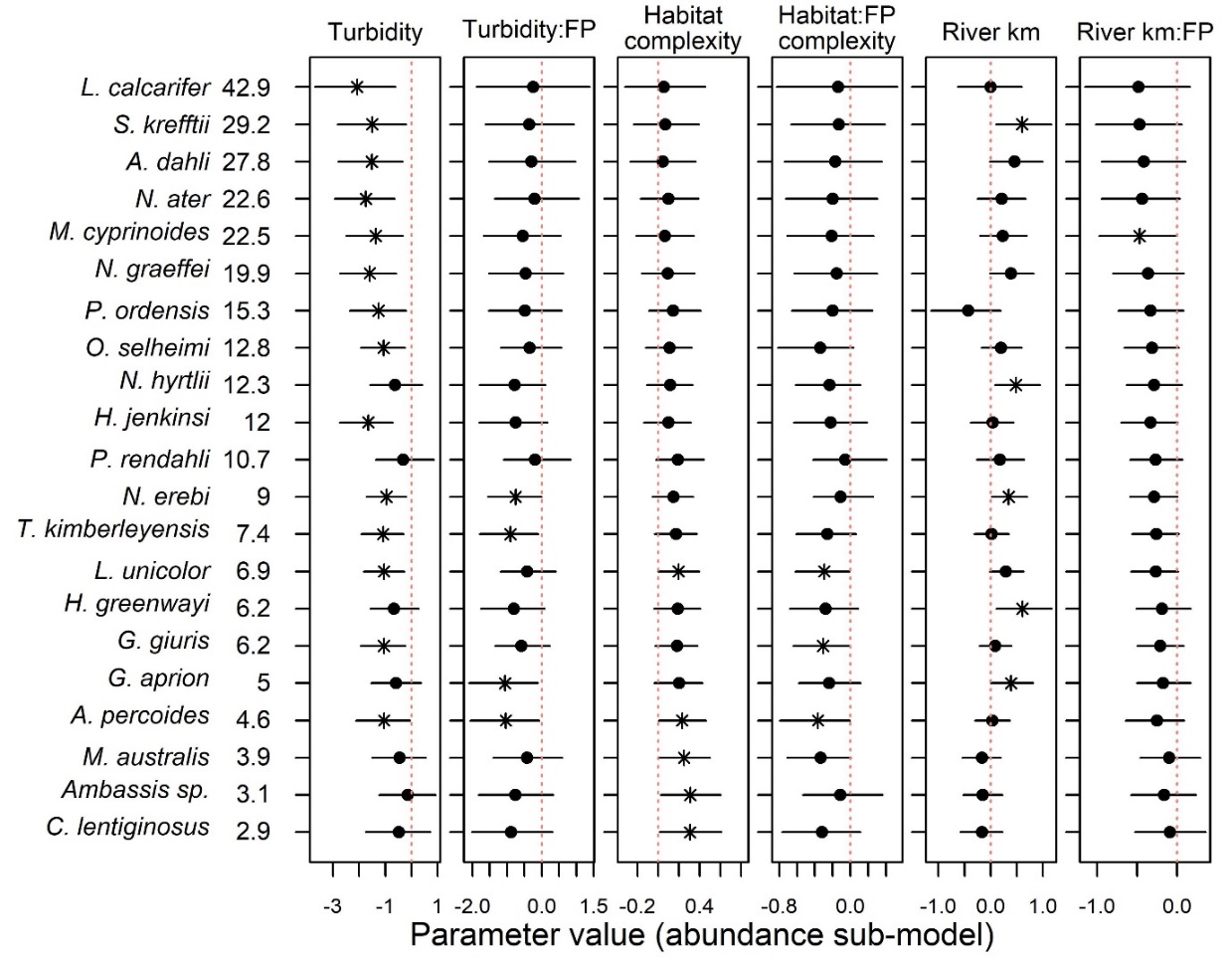


Figure S5. Parameter values for environmental covariates in the abundance sub-model (part two of two). The parameter Turbidity and Turbidity:FP refers to main channel ($\beta_{5,i}$) and floodplain ($\beta_{6,i}$) respectively. Habitat and Habitat:FP complexity refers to the main channel ($\beta_{7,i}$) and floodplain ($\beta_{8,i}$) respectively. Similarly River km and Riverkm:FP refer to the main channel ($\beta_{9,i}$) and floodplain ($\beta_{10,i}$) respectively. Fish species are ordered from largest (top) to smallest (bottom) in accordance with their mean length (cm) collected during the study (length data to the right of their name). The black circular symbol indicates the mean and the whiskers the 90% Bayesian Credible Intervals. The dashed vertical red line indicates an effect of zero and * indicate species where parameter estimates are significant at the alpha 0.01 level (i.e., CI’s do not overlap zero). Beta parameters are shown in equation 1 in the main text.


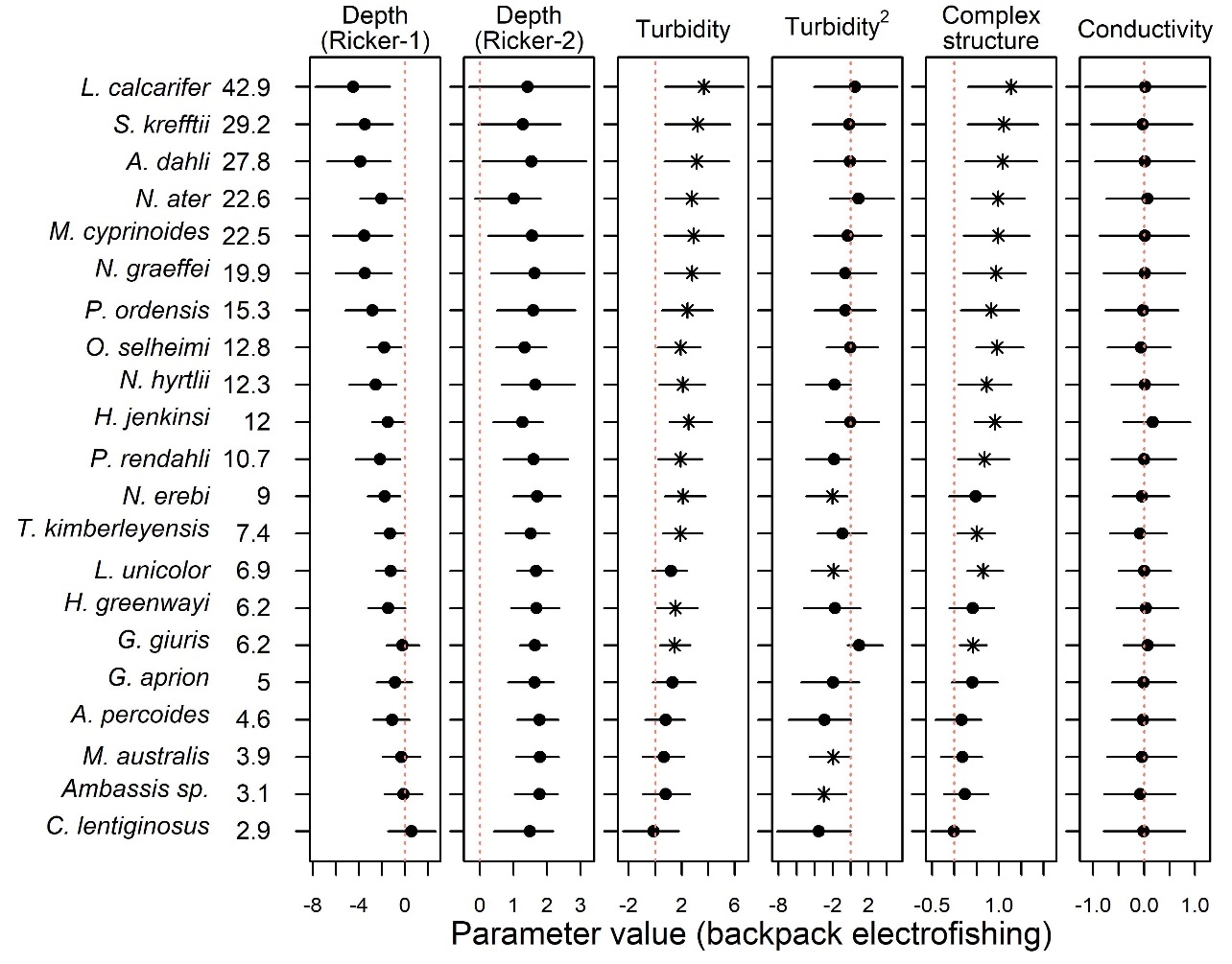


Figure S6. Parameter values for environmental covariates in the detection sub-model describing backpack electrofishing. The parameter Depth (Ricker-1) refers to the first depth parameter in Ricker model $(\varphi_{1,i})$ and Depth (Ricker-2) refers to the second depth parameter in Ricker model $(\varphi_{2,i}$). Turbidity refers to the log turbidity (NTU) ($\varphi_{3,i}$) and Turbidity^2^ the quadratic term ($\varphi_{4,i}$). Complex structure refers to complex structure at the sample location ($\varphi_{5,i}$) and Conductivity to electrical conductivity (μS L^-1^) ($\varphi_{6,i}$). Fish species are ordered from largest (top) to smallest (bottom) in accordance with their mean length (cm) collected during the study (length data to the right of their name). The black circular symbol indicates the mean and the whiskers the 90% Bayesian Credible Intervals. The dashed vertical red line indicates an effect of zero and * indicate species where parameter estimates are significant at the alpha 0.01 level (i.e., CI’s do not overlap zero). Phi parameters are shown in equation 2 in the main text.


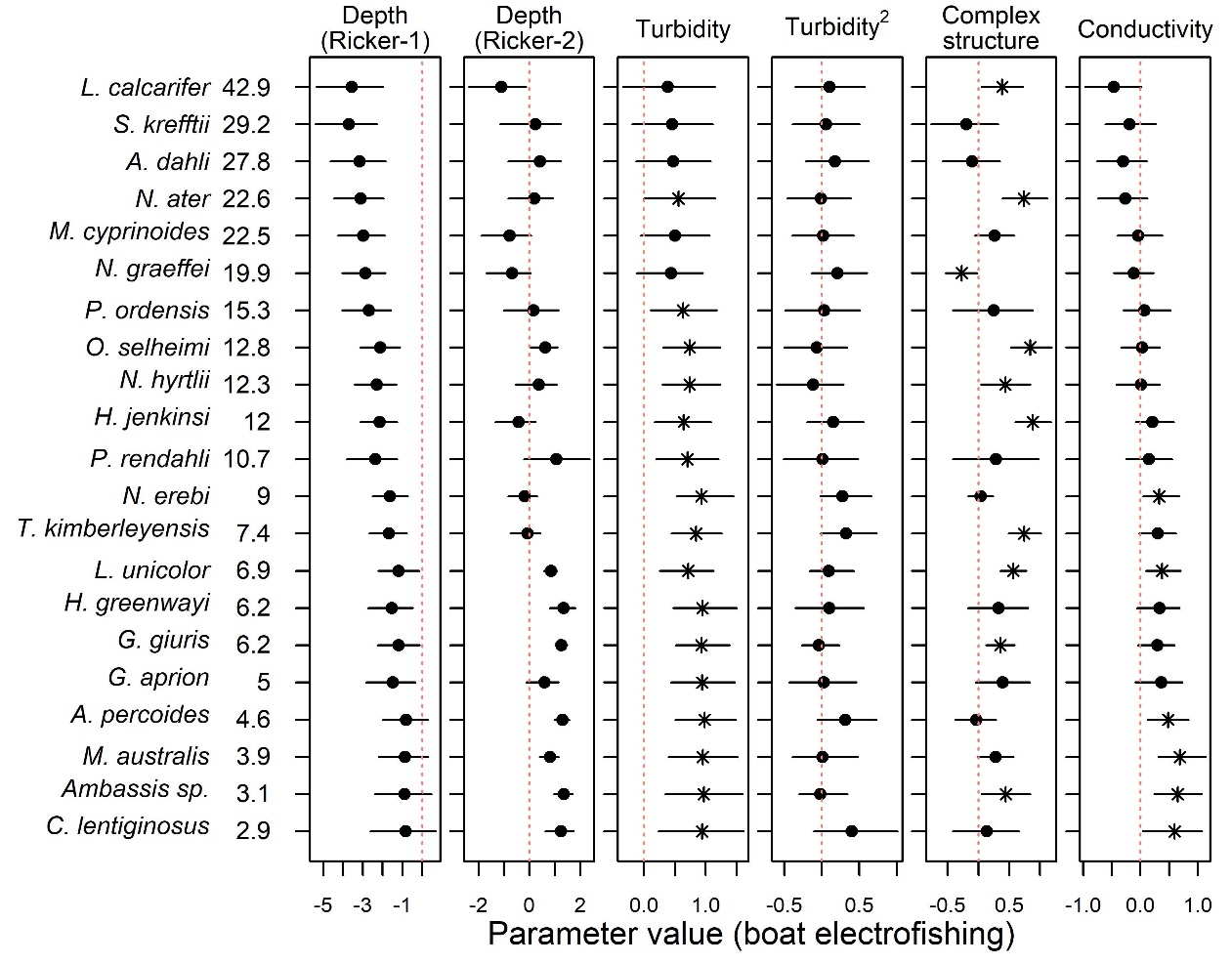


Figure S7. Parameter values for environmental covariates in the detection sub-model describing boat electrofishing. All details are as per Figure S6.


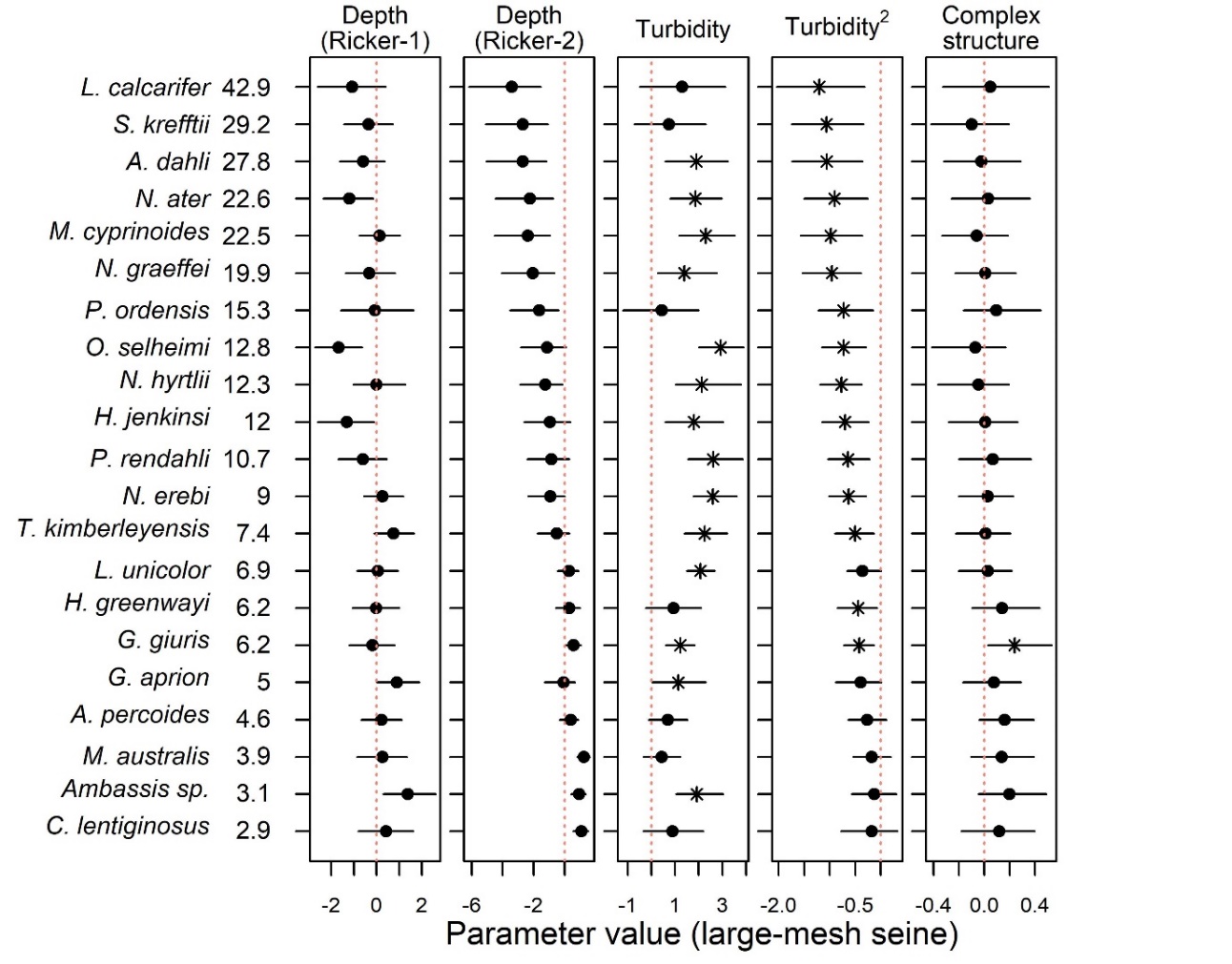


Figure S8. Parameter values for environmental covariates in the detection sub-model describing the large-mesh seine. All details are as per Figure S6.


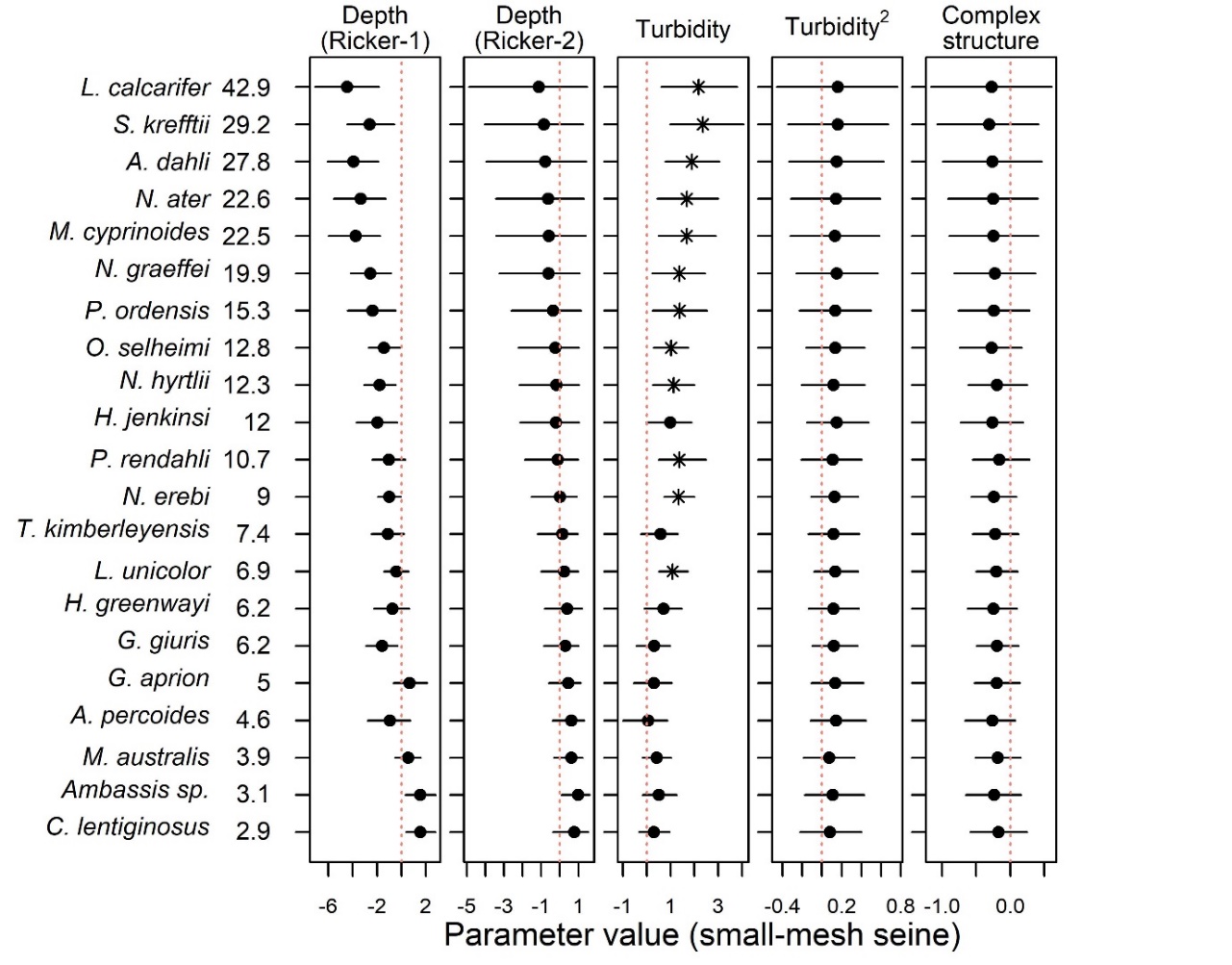


Figure S9. Parameter values for environmental covariates in the detection sub-model describing the small-mesh seine. All details are as per Figure S6.

**Posterior parameter summaries for fixed model terms to investigate the predictive capacity of species length**

The posterior summaries presented in the tables below are pertinent to our investigation of whether the species-level covariate of length can predict the abundance and detection environmental and sampling covariates. The ‘main effects’ presented below refer to parameter $\eta_{1,s}$ in the model (see equation 5 in the main text) and the length effect refers to parameter $\eta_{2,s}$. Note that Figures S4 to 9 (above) only present the ‘main effect’ parameter summaries for simplicity. The parameter names presented in the tables below are as per those presented in the figures above.

Table S4. Posterior parameter summaries for the fixed effects in the abundance sub-model. Bold indicates a statistically significant difference from zero at the $\alpha\leq0.1$.

|  | Main effects | | |  | Length effects | | |
| --- | --- | --- | --- | --- | --- | --- | --- |
| Description | Parameter | $\mu$ | $\sigma$ |  | Parameter | $\mu$ | $\sigma$ |
| Depth (Ricker-1) | bet[1] | 2.609 | 0.391 |  | **bet[11]** | **-0.915** | **0.379** |
| Depth (Ricker-2) | bet[2] | 0.814 | 0.298 |  | bet[12] | -0.362 | 0.251 |
| Depth:FP (Ricker-1) | bet[3] | -0.316 | 0.461 |  | bet[13] | 0.232 | 0.485 |
| Depth:FP (Ricker-2) | **bet[4]** | **0.576** | **0.354** |  | **bet[14]** | **0.588** | **0.335** |
| Turbidity | **bet[5]** | **-1.058** | **0.383** |  | bet[15] | -0.422 | 0.398 |
| Turbidity:FP | bet[6] | -0.586 | 0.379 |  | bet[16] | 0.179 | 0.386 |
| Habitat complexity | bet**[7]** | **0.157** | **0.093** |  | bet[17] | -0.074 | 0.098 |
| Habitat:FP complexity | bet[8] | -0.220 | 0.146 |  | bet[18] | 0.042 | 0.155 |
| River km | bet[9] | 0.178 | 0.129 |  | bet[19] | 0.090 | 0.135 |
| River km:FP | **bet[10]** | **-0.291** | **0.146** |  | bet[20] | -0.112 | 0.158 |

Table S5. Posterior parameter summaries for the fixed effects of the different observation sub-models. Bold indicates a statistically significant difference from zero at the $\alpha\leq0.1$.

|  | Main effects | | |  | Length effects | | |
| --- | --- | --- | --- | --- | --- | --- | --- |
| Description | Parameter | $\mu$ | $\sigma$ |  | Parameter | $\mu$ | $\sigma$ |
|  |  |  |  |  |  |  |  |
| Backpack e-fishing |  |  |  |  |  |  |  |
| Depth (Ricker-1) | bp.phi[1] | **-1.872** | **0.719** |  | bp.phi[7] | **-1.275** | **0.710** |
| Depth (Ricker-2) | bp.phi[2] | **1.542** | **0.362** |  | bp.phi[8] | -0.115 | 0.376 |
| Turbidity | bp.phi[3] | **1.943** | **0.623** |  | bp.phi[9] | 0.942 | 0.683 |
| Turbidity2 | bp.phi[4] | -1.067 | 0.866 |  | bp.phi[10] | 0.944 | 1.038 |
| Complex structure | bp.phi[5] | **0.659** | **0.191** |  | bp.phi[11] | **0.346** | **0.202** |
| Conductivity | bp.phi[6] | 0.004 | 0.256 |  | bp.phi[12] | 0.018 | 0.283 |
|  |  |  |  |  |  |  |  |
| Boat e-fishing |  |  |  |  |  |  |  |
| Depth (Ricker-1) | be.phi[1] | **-2.054** | **0.468** |  | be.phi[7] | **-0.908** | **0.435** |
| Depth (Ricker-2) | be.phi[2] | **0.404** | **0.232** |  | be.phi[8] | **-0.551** | **0.235** |
| Turbidity | be.phi[3] | **0.734** | **0.184** |  | be.phi[9] | -0.191 | 0.199 |
| Turbidity2 | be.phi[4] | 0.100 | 0.113 |  | be.phi[10] | -0.028 | 0.117 |
| Complex structure | be.phi[5] | **0.323** | **0.115** |  | be.phi[11] | -0.042 | 0.117 |
| Conductivity | be.phi[6] | 0.168 | 0.119 |  | be.phi[12] | **-0.313** | **0.125** |
|  |  |  |  |  |  |  |  |
| Large-mesh seine |  |  |  |  |  |  |  |
| Depth (Ricker-1) | lm.phi[1] | -0.142 | 0.389 |  | lm.phi[7] | -0.483 | 0.308 |
| Depth (Ricker-2) | lm.phi[2] | **-0.844** | **0.565** |  | lm.phi[8] | **-1.355** | **0.462** |
| Turbidity | lm.phi[3] | **1.591** | **0.374** |  | lm.phi[9] | 0.144 | 0.386 |
| Turbidity2 | lm.phi[4] | **-0.631** | **0.181** |  | lm.phi[10] | -0.318 | 0.200 |
| Complex structure | lm.phi[5] | 0.053 | 0.075 |  | lm.phi[11] | -0.062 | 0.082 |
|  |  |  |  |  |  |  |  |
| Small-mesh seine |  |  |  |  |  |  |  |
| Depth (Ricker-1) | sm.phi[1] | **-1.453** | **0.573** |  | sm.phi[7] | **-1.585** | **0.511** |
| Depth (Ricker-2) | sm.phi[2] | -0.050 | 0.871 |  | sm.phi[8] | -0.577 | 0.649 |
| Turbidity | sm.phi[3] | **1.078** | **0.317** |  | sm.phi[9] | **0.602** | **0.327** |
| Turbidity2 | sm.phi[4] | 0.129 | 0.138 |  | sm.phi[10] | 0.017 | 0.139 |
| Complex structure | sm.phi[5] | -0.230 | 0.193 |  | sm.phi[11] | -0.022 | 0.196 |

**References**

Gelman, A., and J. Hill. 2006. Data analysis using regression and multilevel/hierarchical models. Cambridge University Press.

Leng, C., M.-N. Tran, and D. Nott. 2014. Bayesian adaptive lasso. Annals of the Institute of Statistical Mathematics **66**:221-244.

Lu, Z., and W. Lou. 2022. Bayesian approaches to variable selection: a comparative study from practical perspectives. The International Journal of Biostatistics **18**:83-108.

Plummer, M. 2003. JAGS: A program for analysis of Bayesian graphical models using Gibbs sampling. Page 125 *in* Proceedings of the 3rd international workshop on distributed statistical computing. Vienna.

Royle, J. A., and J. D. Nichols. 2003. Estimating abundance from repeated presence-absence data or point counts. Ecology **84**:777-790.

Yamaura, Y., J. Andrew Royle, K. Kuboi, T. Tada, S. Ikeno, and S. i. Makino. 2011. Modelling community dynamics based on species-level abundance models from detection/nondetection data. Journal of Applied Ecology **48**:67-75.
